# HemAtlas: A Multi-omics Hematopoiesis Database

**DOI:** 10.1093/gpbjnl/qzaf026

**Published:** 2025-03-19

**Authors:** Zhixin Kang, Tongtong Zhu, Dong Zou, Mengyao Liu, Yifan Zhang, Lu Wang, Zhang Zhang, Feng Liu

**Affiliations:** State Key Laboratory of Membrane Biology, Institute of Zoology, Chinese Academy of Sciences, Beijing 100101, China; Institute for Stem Cell and Regeneration, Chinese Academy of Sciences, Beijing 100101, China; University of Chinese Academy of Sciences, Beijing 100049, China; National Genomics Data Center, China National Center for Bioinformation, Beijing 100101, China; Beijing Institute of Genomics, Chinese Academy of Sciences, Beijing 100101, China; University of Chinese Academy of Sciences, Beijing 100049, China; National Genomics Data Center, China National Center for Bioinformation, Beijing 100101, China; Beijing Institute of Genomics, Chinese Academy of Sciences, Beijing 100101, China; State Key Laboratory of Experimental Hematology, Institute of Hematology and Blood Diseases Hospital, Chinese Academy of Medical Sciences and Peking Union Medical College, Tianjin 300020, China; School of Life Sciences, Shandong University, Qingdao 266000, China; State Key Laboratory of Experimental Hematology, Institute of Hematology and Blood Diseases Hospital, Chinese Academy of Medical Sciences and Peking Union Medical College, Tianjin 300020, China; National Genomics Data Center, China National Center for Bioinformation, Beijing 100101, China; Beijing Institute of Genomics, Chinese Academy of Sciences, Beijing 100101, China; University of Chinese Academy of Sciences, Beijing 100049, China; State Key Laboratory of Membrane Biology, Institute of Zoology, Chinese Academy of Sciences, Beijing 100101, China; Institute for Stem Cell and Regeneration, Chinese Academy of Sciences, Beijing 100101, China; University of Chinese Academy of Sciences, Beijing 100049, China; School of Life Sciences, Shandong University, Qingdao 266000, China

**Keywords:** Hematopoietic database, Multi-omics, Developmental hematopoiesis, Cross-species analysis, *In vitro* hematopoietic stem and progenitor cell

## Abstract

Advancements in high-throughput omics technologies have facilitated a systematic exploration of crucial hematopoietic organs across diverse species. A thorough understanding of hematopoiesis *in vivo* and facilitation of generating functional hematopoietic stem and progenitor cells (HSPCs) *in vitro* necessitate a comprehensive hematopoietic cross-stage developmental landscape across species. To address this need, we developed HemAtlas, a platform designed for the systematic mapping of hematopoiesis both *in vivo* and *in vitro*. HemAtlas features detailed analyses of multi-omics datasets from humans, mice, zebrafish, and HSPC *in vitro* culture systems. Utilizing literature curation and data normalization, HemAtlas integrates various functional modules, allowing interactive exploration and visualization of any collected omics data based on user-specific interests. Moreover, by applying a systematic and uniform integration method, we constructed organ-wide hematopoietic references for each species with manually curated cell annotations, enabling a comprehensive decoding of cross-stage developmental hematopoiesis at the organ level. Of particular significance are three distinctive functions — single-cell cross-stage, cross-species, and cross-model analyses — that HemAtlas employs to elucidate the hematopoietic development in zebrafish, mice, and humans, and to offer guidance on the generation of HSPCs *in vitro*. Simultaneously, HemAtlas incorporates a comprehensive map of HSPC cross-stage development to reveal HSPC stage-specific properties. Taken together, HemAtlas serves as a crucial resource to advance our understanding of hematopoiesis and is available at https://ngdc.cncb.ac.cn/hematlas/.

## Graphical abstract

**Graphical abstract:**
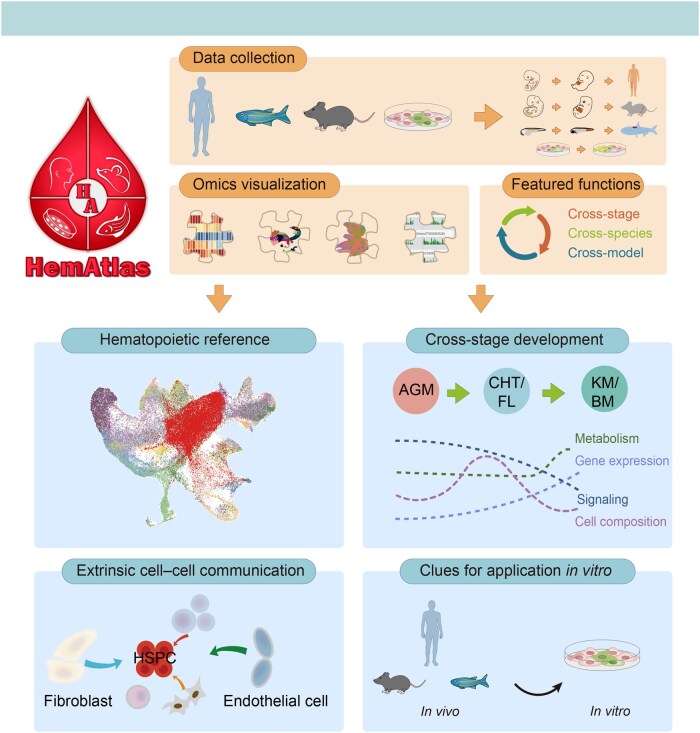

## Introduction

Hematopoietic stem and progenitor cells (HSPCs) maintain a stable pool of hematopoiesis through self-renewal and possess the capacity to generate various blood cells, making them vital for clinical blood disease treatment [1,2]. The development of HSPCs involves key hematopoietic organs. Specifically, HSPCs originate from hemogenic endothelial cells (HECs) in the aorta–gonad–mesonephros (AGM) region. Subsequently, they undergo rapid expansion in the caudal hematopoietic tissue (CHT) in zebrafish or the fetal liver (FL) in mammals before migrating to the kidney marrow (KM) in zebrafish or the bone marrow (BM) in mammals to maintain adult hematopoiesis [3–5]. Recent studies utilizing high-throughput omics technologies, such as single-cell RNA sequencing (scRNA-seq) and spatial transcriptomics, have profiled these critical hematopoietic organs, revealing new regulatory mechanisms in HSPC development [6–11]. However, most studies have focused on individual organs, yet without reconstructing the entire hematopoietic cross-stage development landscape across multiple species.

An alternative approach to overcome this limitation is to gather multi-omics data from diverse hematopoietic organs across various model systems, followed by comprehensive analyses. Currently, several databases, such as BloodSpot [12], ABC portal [13], and StemDriver [14], have been established to compile hematopoiesis-related data. However, BloodSpot is a database that focuses on gene and protein expression data both in normal and malignant hematopoiesis, while ABC portal and StemDriver primarily provide transcriptome data for humans and mice. These resources do not emphasize cross-stage developmental hematopoiesis (*i.e.*, the progression of hematopoiesis from nascent through embryonic to adult stages) across species and lack integration of spatial localization or chromatin accessibility data from multi-omics datasets. Additionally, the development of *in vitro* HSPC culture systems is crucial for generating a sufficient quantity of functional HSPCs for clinical treatments. However, these existing hematopoietic omics databases neither incorporate *in vitro* hematopoietic data nor offer an online tool to guide *in vitro* hematopoiesis. A comprehensive multi-omics hematopoietic database focusing on cross-stage developmental hematopoiesis within commonly used model organisms, while also providing online guidance for *in vitro* HSPC generation, is essential for advancing the exploration of cross-stage and cross-species hematopoietic development and supporting *in vitro* applications.

To address these issues, we introduce a comprehensive multi-omics database, HemAtlas, to map hematopoiesis both *in vitro* and *in vivo*. Leveraging multi-omics datasets from 12 major hematopoietic organs/systems involved in HSPC development, HemAtlas provides a user-friendly visualization platform for exploring standardized datasets and delivers organ-wide hematopoietic references for each species at the single-cell level. Simultaneously, HemAtlas is equipped with three featured functions to uncover hematopoiesis from *in vivo* to *in vitro*. Moreover, with a focus on HSPCs, HemAtlas comprehensively decodes the dynamics of cell state and cell regulation throughout the cross-stage development of HSPCs for each species. Taken together, HemAtlas plays a pivotal role as a valuable hematopoietic database to propel insights into hematopoiesis.

## Database implementation

HemAtlas was developed using Spring Boot (v1.5.16.RELEASE, https://spring.io/projects/spring-boot), a user-friendly framework for creating stand-alone Java applications, as the back-end framework. For creating responsive and visually appealing web pages, the front-end interfaces were constructed using Semantic UI (v2.4, https://semantic-ui.com), a development framework that facilitates the creation of beautiful and responsive layouts with human-friendly HyperText Markup Language (HTML). The browser-based interfaces were coded using Jakarta Server Pages (JSP), jQuery, and Asynchronous JavaScript and Extensible Markup Language (XML) (AJAX), a set of web development technologies that enable the creation of highly interactive web applications, allowing asynchronous data transfer between the server and browser without disrupting the display of the current web page. Additionally, all data visualization charts were generated using ECharts (v5.5, https://echarts.apache.org/), an open-source JavaScript visualization library. For online biological results in cross-model analyses, R Shiny (v1.5.7) was employed. The storage and management of all data were handled through MySQL (v8.0.31, https://dev.mysql.com).

## Database content and usage

### Scheme of HemAtlas

**Figure 1:**
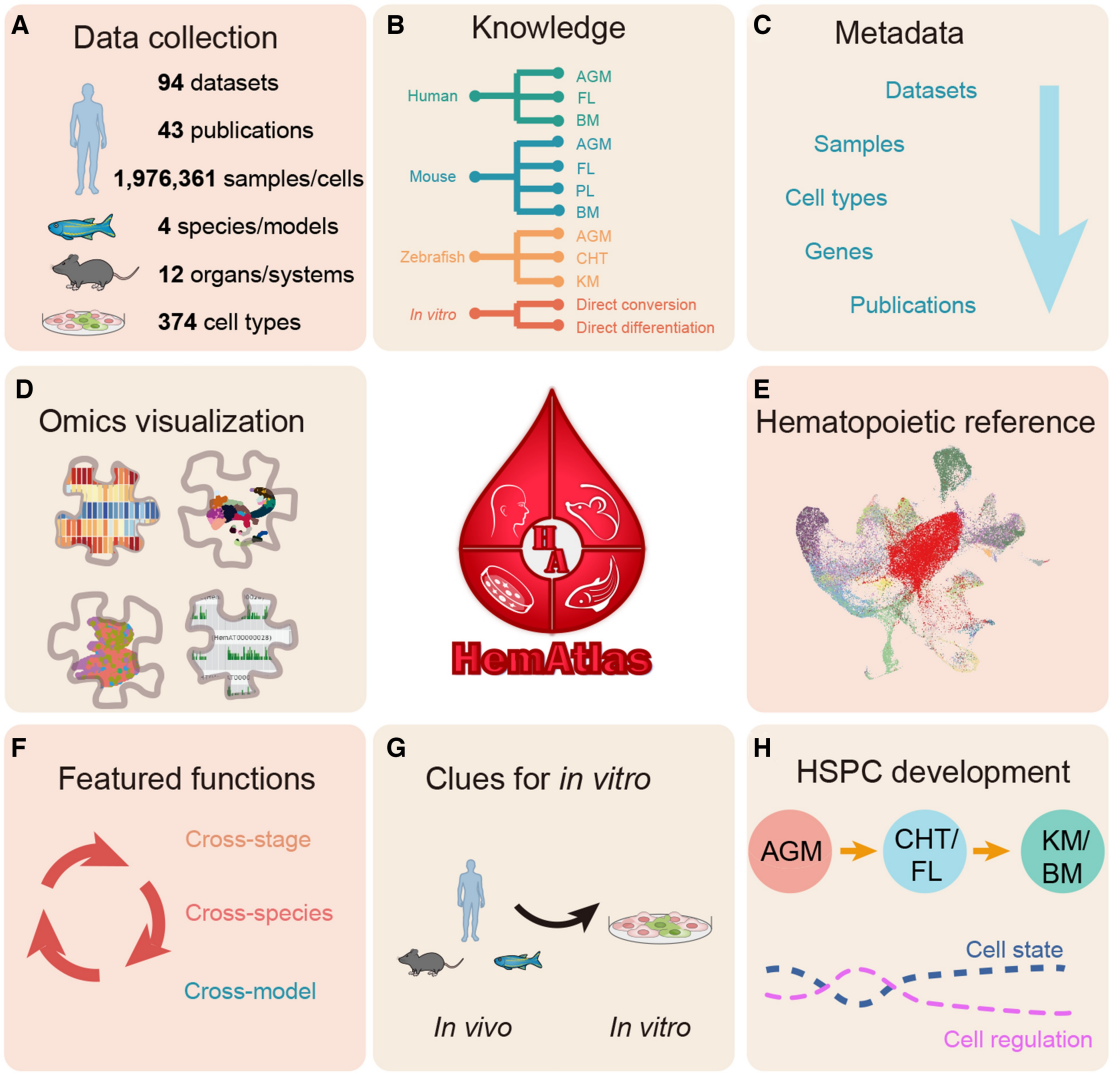
Scheme of HemAtlas **A**. Data statistics of the multi-omics data (including bulk RNA-seq, scRNA-seq, ATAC-seq, scATAC-seq, ChIP-seq, and spatial transcriptomics data) collected in HemAtlas. **B**. Species/models and corresponding stages/systems of collected datasets included in HemAtlas. **C**. Metadata of multi-omics data collected from multiple organs/systems in multiple species/models in HemAtlas. **D**. Visualization of multi-omics data using a four-module platform in HemAtlas. **E**. Organ-wide hematopoietic references constructed for each species. **F**. Three single-cell featured functions deployed in HemAtlas: cross-stage analyses (*i.e.*, online data analyses to reveal the dynamic differences in various stages during hematopoietic development for each species, encompassing distinct cell types, dynamic gene expression, and HSPC states, as well as diverse niche cells and their intercellular communications across different stages), cross-species analyses (*i.e.*, data analyses to unveil the evolutionary conservation and differences in hematopoiesis), and cross-model analyses (*i.e.*, online comparisons between user-uploaded *in vitro* HSPC scRNA-seq data and available *in vivo* omics data to provide insights for engineering the hematopoietic system *in vitro*). **G**. The featured functions in (F) aim to reveal more biological insights into hematopoiesis both *in vitro* and *in vivo*. **H**. The HSPC development module constructed in HemAtlas, which aims to decode HSPC cross-stage development across species. AGM, aorta–gonad–mesonephros; FL, fetal liver; BM, bone marrow; PL, placenta; CHT, caudal hematopoietic tissue; KM, kidney marrow; RNA-seq, RNA sequencing, scRNA-seq, single-cell RNA-seq; ATAC-seq, assay for transposase-accessible chromatin with sequencing; scATAC-seq, single-cell ATAC-seq; ChIP-seq, chromatin immunoprecipitation followed by sequencing; HSPC, hematopoietic stem and progenitor cell.

To construct HemAtlas, we collected multi-omics data from studies that are directly relevant to the developmental hematopoiesis within the classical hematopoietic sites of humans and frequently utilized research models (zebrafish, mice, and HSPC *in vitro* culture systems) (File S1; Table S1). In comparison to other related datasets [13–15], HemAtlas specifically focuses on species-conserved and cross-stage developmental hematopoiesis *in vivo* and HSPC generation *in vitro* (Table S2). HemAtlas currently integrates 94 multi-omics datasets from 43 publications (https://ngdc.cncb.ac.cn/hematlas/publications), encompassing 1,976,361 cells/samples across 374 major cell types (**Figure 1**A–C, Figure S1A and B). We have annotated various blood cells (such as HSPCs, erythroid cells, lymphoid cells, and myeloid cells) as well as non-hematopoietic niche cells (such as endothelial cells, fibroblasts, and mesenchymal cells). Meanwhile, HemAtlas provides a visualized platform based on various sequencing methods, including bulk RNA sequencing (RNA-seq), scRNA-seq, assay for transposase-accessible chromatin with sequencing (ATAC-seq) [including single-cell ATAC-seq (scATAC-seq)], chromatin immunoprecipitation followed by sequencing (ChIP-seq), and spatial transcriptomics (Figure 1D, Figure S1C). Notably, ensuring at least one scRNA-seq dataset available for each hematopoietic organ, we used a systematic and uniform analysis workflow to construct the organ-wide hematopoietic references for each species, facilitating the reuse of these datasets and providing a global view of cross-stage developmental hematopoiesis (Figure 1E). Simultaneously, HemAtlas implements three featured functions to comprehensively understand hematopoiesis from various perspectives (Figure 1F and G). Moreover, HemAtlas integrates a comprehensive HSPC cross-stage development module. Detailed analyses reveal extensive inter-stage heterogeneity in HSPCs, as well as the underlying dynamic gene regulatory networks and cell–cell communications for each species (Figure 1H).

### Interactive exploration and visualization of multi-omics data

**Figure 2:**
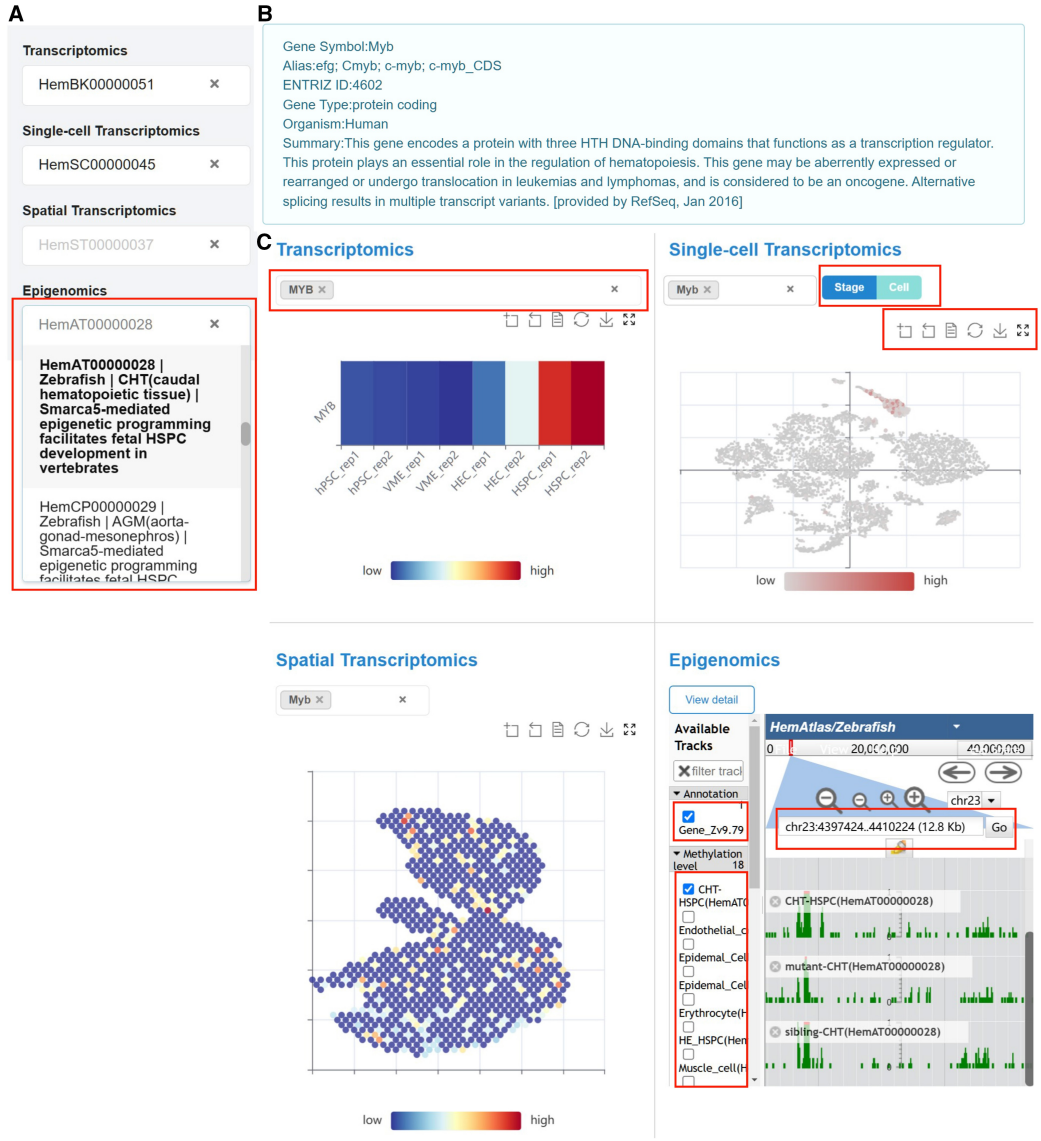
Omics module of HemAtlas **A**. Screenshot of the HemAtlas dataset selection. The red box illustrates a demonstration of the available datasets for the specific omics type (epigenomics). **B**. Detailed description of the gene *MYB* in human. The gene descriptions provided by HemAtlas are based on the RefSeq in the NCBI database (https://www.ncbi.nlm.nih.gov/refseq/). **C**. Upper left: visualization of *MYB* in the selected bulk RNA-seq dataset (HemBK00000051) from HSPC *in vitro* culture system [54]. The red box highlights the region where the gene search was conducted. The color of the bar represents the gene expression level. Upper right: visualization of *Myb* in the selected scRNA-seq dataset (HemSC00000045) from mouse AGM [23]. The upper red box highlights the region where cell annotation based on cell type or stage was conducted, while the lower red box indicates the area where additional online operations can be performed, such as zooming in on the webpage and downloading research results. Lower left: visualization of *Myb* in the selected spatial transcriptomics dataset (HemST00000037) from mouse FL [30]. The color of the bar represents the gene expression level. Lower right: visualization of *myb* in the selected ATAC-seq dataset (HemAT00000028) from zebrafish CHT HSPCs [55]. The red boxes highlight the areas where users can perform additional online operations, such as selecting reference versions, sequencing samples, and choosing genes. NCBI, National Center for Biotechnology Information; RefSeq, Reference Sequence Database.

The primary goal of HemAtlas is to offer researchers an online platform for data exploration tailored to their interests while preserving original analysis results of each dataset to the maximum extent (File S1). To achieve this, following the normalization of multi-omics datasets (File S1), HemAtlas develops a multi-omics visualization module that incorporates four interactive analysis tools for exploring these datasets (**Figure 2**A–C). Taking *MYB* as an example, encoding a key transcription factor (TF) involved in the regulation of hematopoiesis across species [16,17] (Figure 2B), we observed a notable increase in *MYB* expression in the transcriptome dataset from the HSPC *in vitro* culture system, suggesting a potentially crucial role of *MYB* in HSPC generation *in vitro* (Figure 2C). Additionally, *Myb* was found to be enriched in mouse AGM pre-hematopoietic stem cells (pre-HSCs), as demonstrated in the collected single-cell datasets (Figure 2C, Figure S2A–C). Furthermore, for each scATAC-seq dataset, we computed TF activity (the regulatory impact that a TF exerts on the expression of each of its target genes [18]) and observed a substantial increase in *myb* activity in zebrafish HECs and nascent HSPCs (Figure S2D). These findings are consistent with its pivotal role in HSPC generation *in vivo* [19,20]. Other dimensional information, such as the gene’s spatial location in mouse FL and gene accessibility in zebrafish CHT HSPCs, can be simultaneously accessed (Figure 2C). Users can explore the details of these datasets by clicking on the zoom button in the upper right of each dataset (Figure 2C, Figure S2A–D). In summary, the omics module in HemAtlas provides a valuable platform for the interactive exploration and visualization of multi-omics data based on users’ interests (https://ngdc.cncb.ac.cn/hematlas/omics/) with original analysis results.

### Construction of organ-wide hematopoietic references based on scRNA-seq data

**Figure 3:**
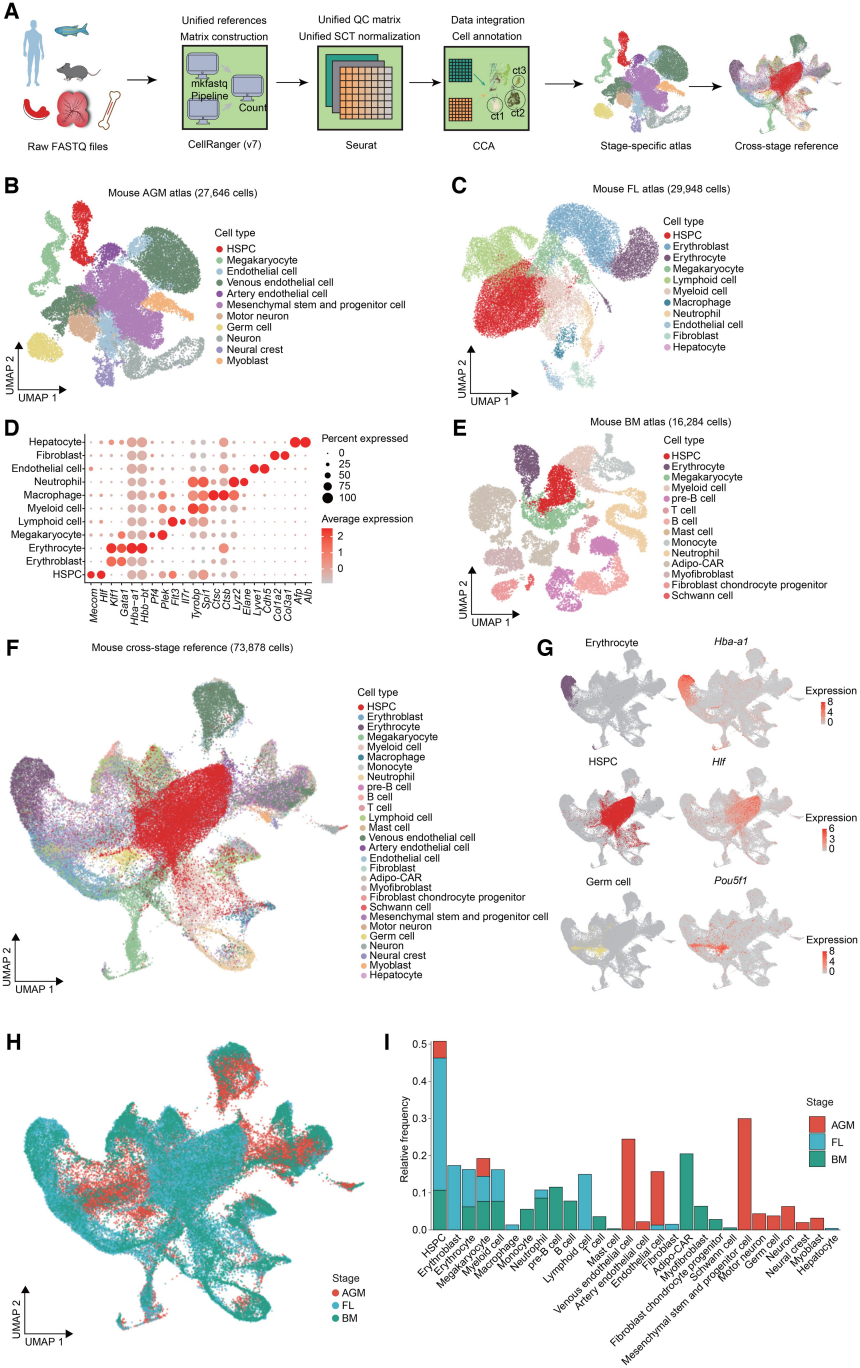
Organ-wide hematopoietic references based on scRNA-seq data in mice **A**. Workflow for constructing organ-wide hematopoietic references, including both stage-specific atlases and cross-stage references, for each species. **B**. Visualization of mouse AGM atlas, which includes 27,646 cells and 11 major cell types. Cells are color-coded by their annotated cell types. **C**. Visualization of mouse FL atlas, which includes 29,948 cells and 11 major cell types. Cells are color-coded by their annotated cell types. **D**. Dot plot showing the expression of marker genes for cell annotation in mouse FL atlas. The size of the dot corresponds to the percentage of cells expressing the gene. The color represents the average expression level. **E**. Visualization of mouse BM atlas, which includes 29,948 cells and 14 major cell types. Cells are color-coded by their annotated cell types. **F**. Visualization of mouse cross-stage hematopoietic reference, which includes 73,878 cells and 28 major cell types across 3 developmental stages (AGM, FL, and BM). Cells are color-coded by their annotated cell types. **G**. UMAP visualization of the representative mouse cell types and scaled expression of the corresponding cell type-specific marker genes in the mouse cross-stage hematopoietic reference. Cells are color-coded by their annotated cell types in (F) and scaled gene expression levels, respectively. **H**. Visualization of mouse cross-stage hematopoietic reference. Cells are color-coded by the stages to which they belong. **I**. Cell number statistics in the mouse cross-stage hematopoietic reference across three developmental stages (AGM, FL, and BM). The colors represent corresponding developmental stages in (H). QC, quality control; SCT, SCTransform; CCA, canonical correlation analysis; ct, cell type; FASTQ, Fast Quality; UMAP, Uniform Manifold Approximation and Projection; Adipo-CAR, Adipo-Cxcl12-abundant-reticular.

To comprehensively decode cross-stage developmental hematopoiesis at organ levels and facilitate better reuse of the collected scRNA-seq datasets, we constructed organ-wide hematopoietic references in each species at single-cell resolution using a step-by-step and standardized analysis workflow for each species (**Figure 3**A). First, we downloaded the raw Fast Quality (FASTQ) files from each dataset (refer to File S1 for details on the datasets used and their selection criteria). We then employed the unified CellRanger pipeline to generate expression matrices by mapping to the same reference transcriptome for each species (human: GRCh38; mouse: mm10; zebrafish: GRCz11). Following this, we conducted unified quality control on each dataset and normalized the raw counts using the widely accepted SCTransform method in Seurat [21] (File S1). For each stage within each species, we integrated data from individual datasets to construct stage-specific atlases (Figure 3B–E, Figures S4A–D and S5A–D). For example, two re-analyzed datasets from mouse AGM [22,23] were used to construct the mouse AGM atlas HemSC01000088 (https://ngdc.cncb.ac.cn/hematlas/dataset/HemSC01000088). We then aggregated these stage-specific atlases across all stages to develop cross-stage references for each species (Figure 3F–I, Figures S4E–H and S5E–H). For example, the mouse AGM atlas HemSC01000088, mouse FL atlas HemSC01000089, and mouse BM atlas HemSC01000090 were combined to construct the mouse cross-stage reference HemSC01000091 (https://ngdc.cncb.ac.cn/hematlas/dataset/HemSC01000091). All cell types in these organ-wide hematopoietic references — both stage-specific atlases and cross-stage references — were manually re-annotated and standardized (Figure 3D, Figures S4B and S5D; Table S3; File S1). It should be noted that many of the collected datasets were highly enriched for HSPCs, such as those from zebrafish AGM [24] and CHT [8]. As a result, we re-annotated many HSPCs based on the expression of well-known HSPC marker genes in these organ-wide hematopoietic references (Figure 3F, Figures S4E and S5E; Table S3; File S1). Moreover, as epigenome and spatial transcriptome datasets were occasionally unavailable for specific stages, cross-stage integration was not conducted for these datasets (File S1). However, it is important to highlight that we can cross-validate the analysis results of scRNA-seq data in these omics datasets.

Various integration algorithms have different integration performance to correct the non-biological information [25]. To compare the performance of different integration methods in constructing organ-wide hematopoietic references, we performed Local Inverse Simpson’s Index (LISI) analyses [26], evaluating methods including mutual nearest neighbors (MNN) [27], Harmony [26], and canonical correlation analysis (CCA) method [28], during the construction of the mouse cross-stage hematopoietic reference (Figure S3A; File S1). The results demonstrated that the CCA method we used outperformed other methods in integrating datasets from different stages (Figure S3B) and, importantly, was the only method that successfully grouped mouse *Hlf*-positive cells (HSPCs) together (Figure 3G, Figure S3C). To further assess the potential batch effects after CCA correction in the mouse cross-stage reference, we performed the k-nearest neighbor batch effect test (kBET) analyses both before and after data correction [29]. The significantly lower kBET values after CCA correction demonstrated that our method effectively mixed different datasets (batches) together after data correction (Figure S3D). Together, these findings suggest that CCA is highly effective for constructing organ-wide hematopoietic references (further details refer to File S1).

The resulting nine stage-specific atlases capture major cell types across the three critical development stages of hematopoiesis in each species, including both stage-common cell types such as HSPCs, macrophages, and neutrophils, and stage-specific types such as urogenital ridge cells, hepatocytes, and myofibroblasts (Figure 3B–E, Figures S4A–D and S5A–D). Researchers can explore cell composition, gene expression, and cell–cell communication in specific developmental stages and species using these constructed stage-specific atlases, such as zebrafish AGM (Figure S4A and B), mouse FL (Figure 3C and D), and human BM (Figure S5C and D) based on their interested biological questions (refer to File S1 for a discussion on the impact of cell enrichment on cell composition and cell–cell communication analyses).

Moreover, the systematically integrated cross-stage references, constructed using consistent standards and workflows, offer a uniform and comprehensive view of species-conserved developmental hematopoiesis across stages. Specifically, the zebrafish cross-stage reference includes a total of 62,954 cells (Figure S4E), while the mouse and human cross-stage references comprise 73,878 and 242,899 cells, respectively (Figure 3F, Figure S5E). These references facilitate direct cross-stage analyses, enabling researchers to explore gene expression (Figure 3F and G, Figures S4E and F and S5E and F) and cell composition (Figure 3H and I, Figures S4G and H and S5G and H) across different stages for each species in HemAtlas. For instance, in the subsequent cross-stage analyses, we conducted a comprehensive examination of the dynamic expression of *csf1ra* and *csf1rb* in zebrafish using the zebrafish cross-stage reference.

To assist researchers in exploring these comprehensive organ-wide hematopoietic references, we developed an online module in HemAtlas for interactive exploration and visualization (https://ngdc.cncb.ac.cn/hematlas/references). Additionally, we provided a detailed description in the frequently asked questions (FAQ) section (https://ngdc.cncb.ac.cn/hematlas/faq) on the HemAtlas website to guide users on how to further explore these references online. Importantly, researchers can also directly download these pre-processed references from HemAtlas for personalized analyses based on specific scientific questions, such as cell annotation and differential analyses (https://ngdc.cncb.ac.cn/hematlas/download/). For instance, in our organ-wide hematopoietic references, we annotated numerous HSPCs, including both hematopoietic stem cells (HSCs) and hematopoietic progenitor cells (HPCs). Users can download these datasets to perform further analyses, such as further identifying HSCs and HPCs or exploring HSPC subclusters with distinct transcriptomic features [8,30].

### Mapping hematopoiesis across stages, species, and models

**Figure 4:**
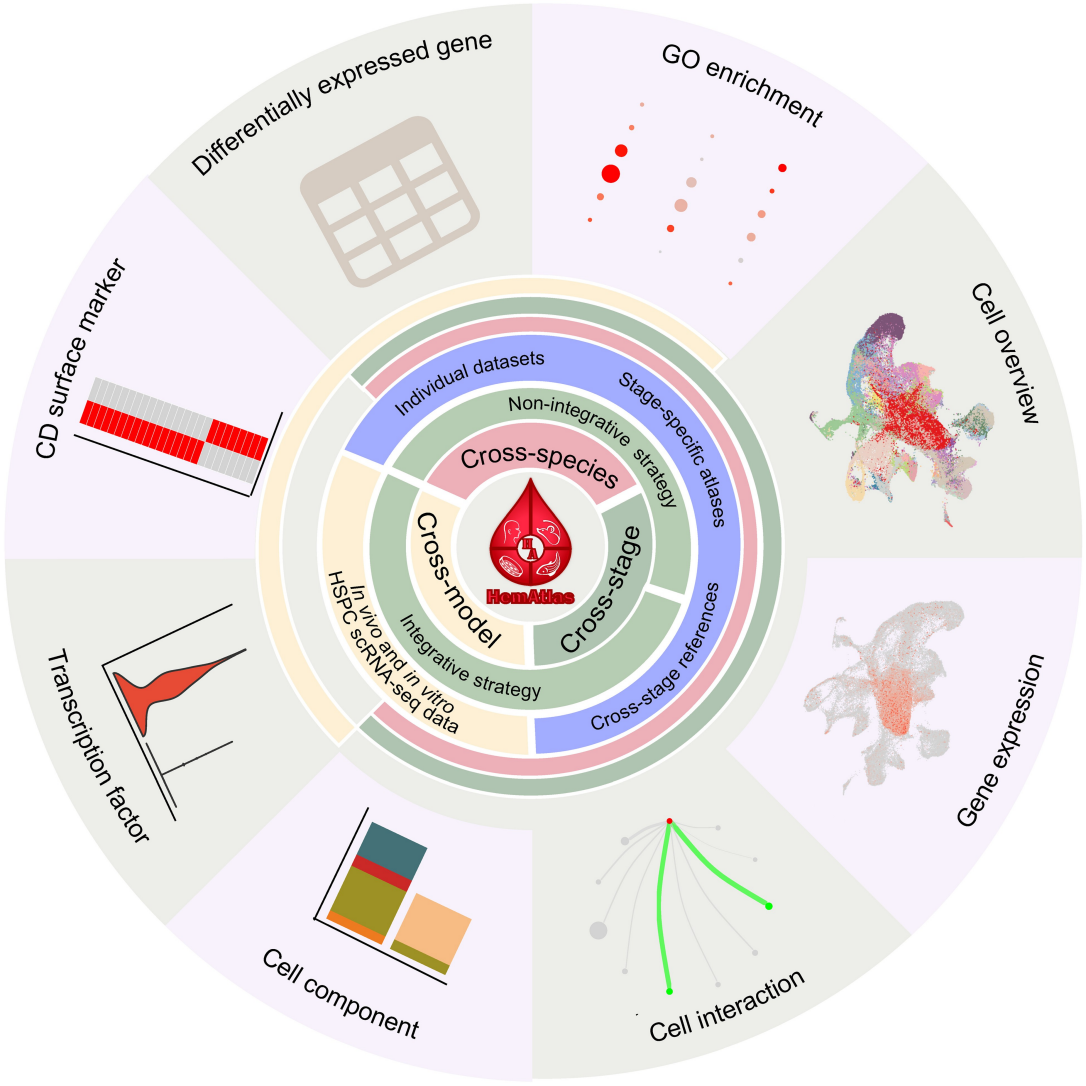
Featured functions in HemAtlas A summary of the cross-dataset comparison strategies, the datasets used, and the various online analysis tools within the three featured functions in HemAtlas. In the cross-stage analysis function of HemAtlas, researchers can utilize both non-integrative and integrative strategies to perform comprehensive cross-dataset analyses online. The available datasets include individual datasets, stage-specific atlases, and cross-stage references. The online analysis tools available for cross-stage and cross-species analyses encompass cell overview, gene expression, cell component, differentially expressed gene, GO enrichment, and cell interaction. Meanwhile, the cross-model analyses use integrative strategy and primarily comprise two sections: identification of HSPC similarities between *in vitro* and *in vivo* and identification of differentially key factors, such as TFs and CD surface factors, across distinct modules (*in vitro* and *in vivo*) for HSPCs. GO, Gene Ontology; TF, transcription factor; CD, cell “differentiation”.

Hematopoiesis is a species-conserved process encompassing various developmental stages [1,2]. To comprehensively understand hematopoiesis across stages and evolution, and provide guidance for obtaining HSPCs with clinical potential *in vitro*, HemAtlas features three user-friendly functions designed for online cross-dataset comparison (**Figure 4**).

#### Two strategies for cross-dataset comparison

Cross-dataset comparisons across stages, species, and models are inherently complex due to the influence of various non-biological variations, which often necessitate systematic data integration. However, such integration can risk losing the original analysis results of individual datasets [13]. To address this challenge, we leveraged individual scRNA-seq datasets and organ-wide hematopoietic references to develop two strategies (non-integrative strategy and integrative strategy) for cross-dataset comparison (Figure 4; File S1). These strategies aim to balance the retention of original analysis results while facilitating systematic cross-dataset analyses.

The non-integrative strategy was employed for cross-stage and cross-species analyses. Researchers can use both the individual scRNA-seq datasets (which preserve the original analysis results of each dataset) and stage-specific atlases to perform cross-dataset comparison without the need for data integration across stages and species. Multiple user-interesting datasets will be displayed on a single webpage, and various analysis modules, such as cell overview, gene expression, cell component, differentially expressed gene (DEG), Gene Ontology (GO) enrichment, and cell interaction, can be used to facilitate cross-dataset comparisons dataset by dataset [15,31].

The integrative strategy was applied in cross-stage and cross-model analyses (Figure 4). Researchers can utilize constructed cross-stage references for each species, which involve systematic data integration and manual cell re-annotation, to perform direct cross-stage comparison, such as gene expression and cell composition dynamics across stages in a single integrated dataset. Additionally, in cross-model analyses, *in vitro* and *in vivo* datasets are integrated to identify similarities and differences between the two models using integrative strategy.

The detailed methodologies and applications of these strategies in each featured function are explained in the sections below and File S1.

#### Cross-stage analyses

This function aims to unveil the developmental hematopoiesis across different stages in each species. Currently, for each species, canonical HSPC-involved hematopoietic organs (AGM, CHT/FL, and KM/BM) have been incorporated. The cross-stage analyses support both the non-integrative strategy and the integrative strategy.

**Figure 5:**
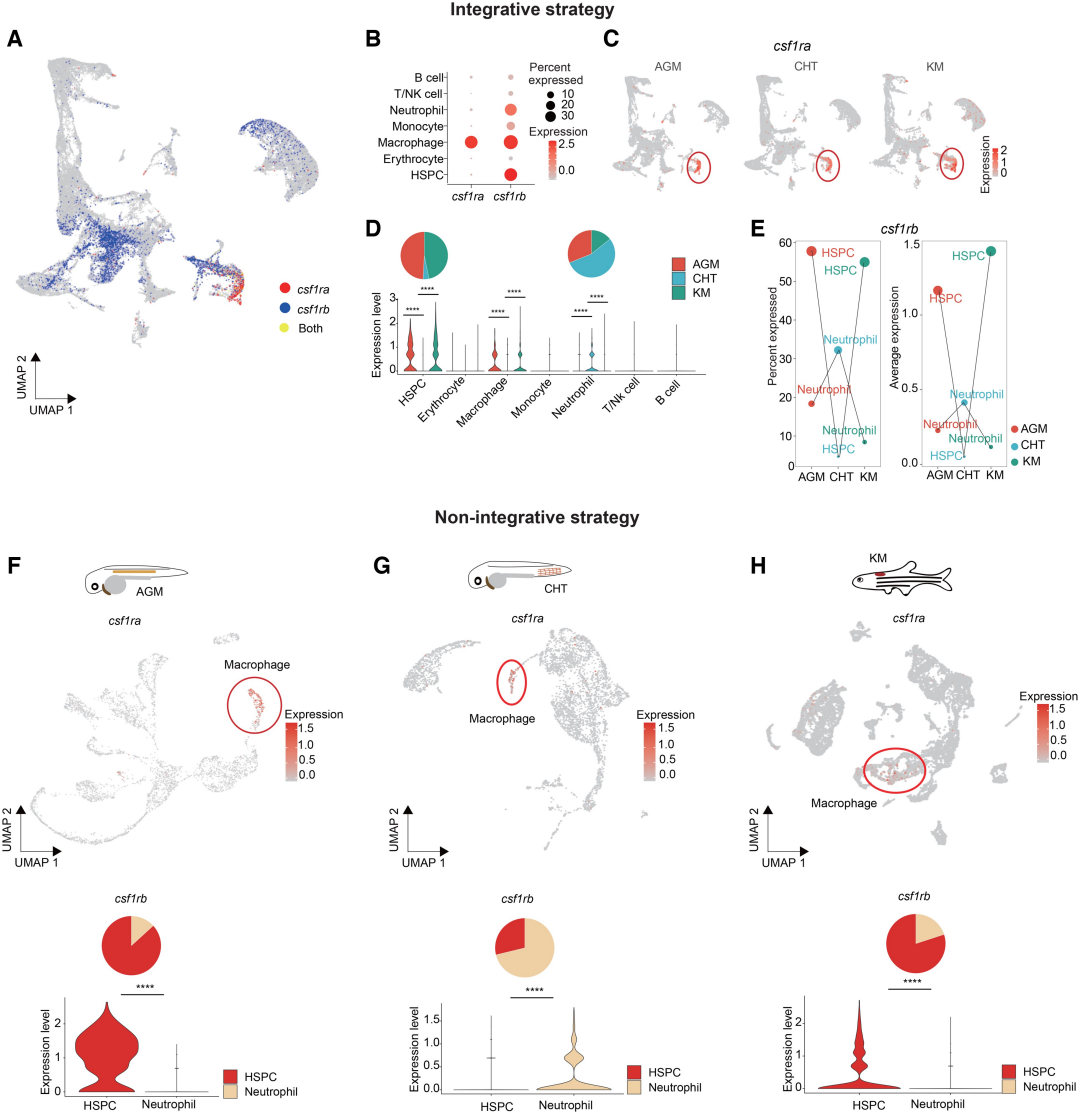
Cross-stage analyses of *csf1ra* and *csf1rb* in zebrafish using two strategies **A**. Visualization of the expression of *csf1ra* and *csf1rb* in the constructed zebrafish cross-stage reference. Cells are color-coded based on the expression levels of *csf1ra* and *csf1rb*. “*csf1ra*” represents cells with normalized *csf1ra* expression levels greater than 0, and “*csf1rb*” represents cells with normalized *csf1rb* expression levels over 0. “Both” indicates cells where the normalized expression levels both *csf1ra* and *csf1rb* exceed 0. **B**. Dot plot illustrating the expression of *csf1ra* and *csf1rb* across different blood cell types in the zebrafish cross-stage reference. Dot size represents the percentage of cells expressing the gene, while color indicates the average expression level. **C**. Visualization of *csf1ra* expression in the constructed zebrafish cross-stage reference across three developmental stages (AGM, CHT, and KM). Cells are color-coded by scaled expression of *csf1ra*, with the red circle highlighting the specific expression of *csf1ra* in macrophages across three developmental stages. **D**. Violin plot depicting the expression levels of *csf1rb* in different blood cell types of zebrafish cross-stage reference across zebrafish developmental stages. Significant differences (****, *P* < 0.0001, *t*-test) were observed in HSPCs, macrophages, and neutrophils between the AGM and CHT stages, as well as between the CHT and KM stages. For HSPCs and neutrophils, the relative proportion of *csf1rb*-positive cells (*i.e.*, the number of cells with normalized *csf1rb* expression levels over 0 *vs.* the total number of cells at each stage for a specific cell type) between three developmental stages is shown in the upper pie chart. **E**. Dot plots showing the percentage of cells expressing *csf1rb* (left) and the average expression level of *csf1rb* (right) across zebrafish AGM, CHT, and KM in HSPCs and neutrophils of zebrafish cross-stage reference. **F**.–**H**. Top: UMAP visualization of *csf1ra* expression in individual datasets from zebrafish AGM [24] (F), CHT [36] (G), and KM [37] (H). The red circles indicate the specific expression of *csf1ra* in macrophages across the three developmental stages. Bottom: violin plot showing the expression levels of *csf1rb* in HSPCs and neutrophils across zebrafish developmental stages in individually selected datasets (****, *P* < 0.0001, *t*-test). Meanwhile, the relative proportion of *csf1rb*-positive cells (*i.e.*, the number of cells with normalized *csf1rb* expression levels over 0 *vs.* the total number of cells in each cell type at a specific stage) between HSPCs and neutrophils is shown in the upper pie chart. NK, natural killer.

For instance, by employing an integrative strategy to analyze cell types from the zebrafish cross-stage reference (Figure S4E and G), we identified macrophages as a stage-common cell type across zebrafish AGM, CHT, and KM (Figure S4H). Previous studies have emphasized the crucial role of the gene *Csf1r* in mouse macrophage development [32,33]. Utilizing the zebrafish cross-stage reference that we constructed, we examined the expression of its paralogous genes, *csf1rb* and *csf1ra*, during zebrafish cross-stage development (**Figure 5**A, Figure S4E). We identified distinct expression patterns for *csf1rb* and *csf1ra*. Specifically, *csf1ra* showed a specific and uniform expression in zebrafish macrophages (Figure 5A and B), whereas *csf1rb* displayed a more variable pattern, with prominent expression in macrophages, HSPCs, and neutrophils, but less in niche cells (Figure 5A and B) [33–35].

Next, we investigated the dynamic expression profiles of *csf1ra* and *csf1rb* across three developmental stages using the integrative strategy in the constructed zebrafish cross-stage reference. We found that *csf1ra* exhibited consistent and specific expression in macrophages throughout the zebrafish AGM, CHT, and KM stages (Figure 5C). In contrast, *csf1rb* displayed a dynamic expression profile across these stages especially in HSPCs and neutrophils (Figure 5D). In the AGM stage, *csf1rb* was highly expressed in HSPCs [34] and macrophages, but not in neutrophils. During the CHT stage, its expression was higher in neutrophils but lower in HSPCs and macrophages. By the KM stage, *csf1rb* was again highly expressed in HSPCs [35] and macrophages [35], with reduced expression in neutrophils (Figure 5D and E). These dynamic changes in *csf1rb* expression across specific cell types may account for the varied gene regulation and function observed at different developmental stages, including HSPCs and HSC-derived myelopoiesis [33,34].

Moreover, researchers can also perform cross-stage analyses using the non-integrative strategy provided by HemAtlas, which preserves the original analysis results of each dataset, in addition to the integrative strategy. When analyzing individual datasets from zebrafish AGM, CHT, and KM stages (specifically, HemSC00000059 [24], HemSC00000048 [36], and HemSC00000043 [37]) using this non-integrative strategy, the dynamic expression profiles of *csf1ra* and *csf1rb* were also revealed in a cross-stage manner (Figure 5F–H) [https://ngdc.cncb.ac.cn/hematlas/featured/organs/compare?species=Zebrafish&organs=AGM(aorta-gonad-mesonephros),Bone%20marrow,Kidney,CHT(caudal%20hematopoietic%20tissue),Fetal%20liver&datasets=HemSC00000043,HemSC00000048,HemSC00000059].

In summary, the two strategies for cross-dataset analyses (*i.e.*, integrative and non-integrative strategies) that we deployed in HemAtlas achieve a balance between retaining original analysis results and the need for consistent data integration. Both strategies provide insights into alterations in cell types, gene expression, and cell regulation during developmental hematopoiesis (https://ngdc.cncb.ac.cn/hematlas/featured/organs). The detailed process of the cross-stage analyses of *csf1ra* and *csf1rb* in zebrafish is provided in the FAQ section on the HemAtlas website (https://ngdc.cncb.ac.cn/hematlas/faq).

#### Cross-species analyses

This function aims to unveil the evolutionary conservation and differences in hematopoiesis. To preserve non-homologous genes, cross-species analyses currently support the non-integrative strategy. Similar to the non-integrative strategy of cross-stage analyses, users can employ various online analysis modules after selecting stages, species, and datasets (including constructed stage-specific atlases and individual scRNA-seq datasets) of interest to perform cross-species analyses (Figure 4; File S1).

##### Case study*:* identifying species-conserved hematopoietic regulatory function of fibroblasts using cross-species analyses

Cells do not live in a vacuum but within a complex microenvironment [38]. Previous studies have demonstrated that various niche cells play a crucial role in regulating cell state and cell fate of HSPCs [8,23,39]. During HSPC cross-stage development in zebrafish and mammals, despite architectural differences such as distinct niche cells and secreted factors [6,30,36], both the CHT in zebrafish and the FL in mammals play crucial roles in supporting HSPC expansion and differentiation during early development. Investigating why the zebrafish CHT and mammalian FL have different architectures but similar functions in supporting HSPC development will provide valuable insights and enhance our understanding of the roles that these tissues play in hematopoiesis.

**Figure 6:**
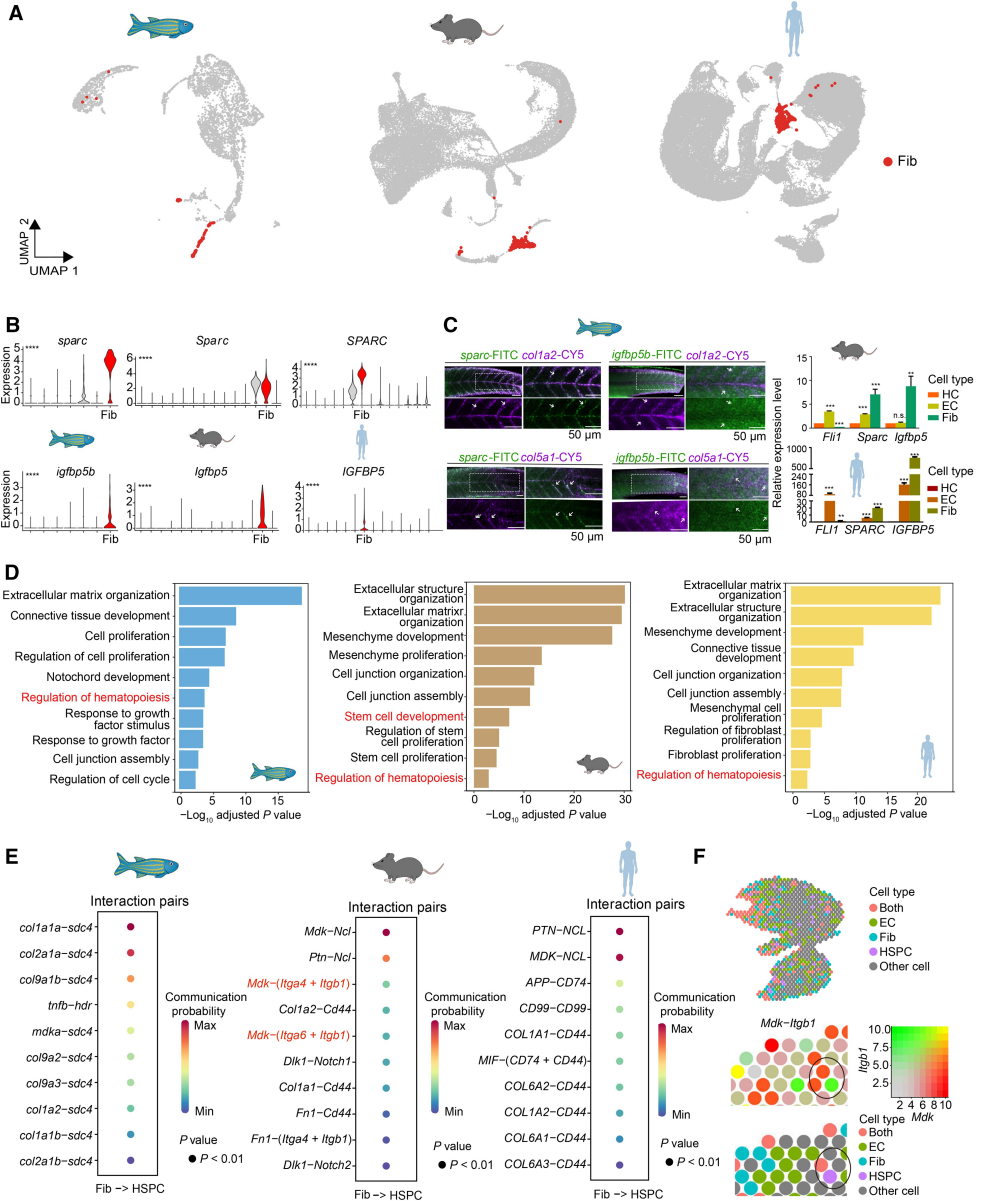
Interactive cross-species analyses of HemAtlas **A**. Visualization of fibroblasts in selected datasets from zebrafish CHT [36], mouse FL [30], and human FL [6], respectively. **B**. Violin plots showing the expression of *sparc/Sparc/SPARC* and *igfbp5b/Igfbp5/IGFBP5* in all cell types from zebrafish CHT, mouse FL, and human FL, respectively, with the fibroblasts highlighted in red. The Kruskal–Wallis test was used to calculate the statistical significance across all the cell types in each dataset (****, *P* < 0.0001). **C**. Left: co-expression of *sparc* and *igfbp5b* with fibroblast markers *col1a2* and *col5a1* is observed in zebrafish CHT fibroblasts (arrowheads) by FISH. Scale bar, 50 μm. Right: quantitative real-time PCR showing that *Sparc/SPARC* and *Igfbp5/IGFBP5* are expressed in fibroblasts of mouse and human FL (*n* = 3). Data are presented as mean ± SEM. Statistical difference was determined by Student’s *t*-test (n.s., not significant; *, *P* < 0.05; **, *P *< 0.01; ***, *P* < 0.001). **D**. GO enrichment analyses showing the conserved fibroblast function across three species. The GO terms related to the regulation of hematopoiesis are highlighted in red. The order of the GO terms is based on the value of −log_10_ adjusted *P* value. **E**. Representative ligand–receptor pairs with the top 10 highest communication probabilities between fibroblasts as ligand-expressing cells and HSPCs as receptor-expressing cells in expanded hematopoietic organs of three species. The order of the ligand–receptor pairs is based on their communication probabilities. **F**. Visualization of the *Mdk*–*Itgb1* interaction pair between HSPCs and fibroblasts in mouse FL spatial transcriptome datasets [40]. Spots are color-coded by cell types and scaled gene expression of *Mdk* and *Itgb1*, respectively. “Both” represents spots that include both fibroblasts and ECs. Circle represents a closer spatial relationship between HSPCs and fibroblasts. FISH, fluorescence *in situ* hybridization; PCR, polymerase chain reaction; SEM, standard error of the mean; Fib, fibroblast; HC, hematopoietic cell; EC, endothelial cell; Max, maximum; Min, minimum.

Based on the stage-specific atlases across three species, fibroblasts were identified as species-conserved niche cells of HSPCs both in zebrafish CHT and mammalian FL (Figure 3C, Figures S4C and S5B). Additionally, analyses of individual scRNA-seq datasets (preserving original analysis results) also confirmed the presence of fibroblasts in zebrafish CHT, mouse FL, and human FL (**Figure 6**A) (the selected datasets: HemSC00000048 [36], HemSC00000041 [30], and HemSC00000017 [6] in https://ngdc.cncb.ac.cn/hematlas/featured/species).

To explore the conservation of fibroblast characteristics, such as gene expression, during evolution and understand their regulatory role in HSPCs, we employed the analysis modules in HemAtlas to perform the cross-species analyses of fibroblasts in three species (non-integrative strategy). Firstly, using the pre-calculated online differential gene analysis module, we identified two top-ranked (with log_10_ fold change > 0.5), conserved fibroblast-specific DEGs across the three species: *sparc/Sparc/SPARC* and *igfbp5b/Igfbp5/IGFBP5* (Figure 6B). The specific expression of these genes in fibroblasts, which had not been previously reported in fibroblasts, was confirmed through validation assays, demonstrating their specific enrichment in fibroblasts across all three species (Figure 6C; Table S4). These findings highlight HemAtlas’s ability to uncover novel, conserved cell type-specific marker genes through cross-species analyses.

Concerning cell function, results from the GO analysis module (File S1) indicated that fibroblasts exhibit a conserved hematopoietic regulatory function besides their traditional function (Figure 6D). This observation is supported by the ligand–receptor pairs identified between fibroblasts and HSPCs in the cell interaction module (Figure 6E), suggesting that HSPCs can be regulated by fibroblasts through cell–cell communications, exemplified by pairs such as *Mdk*–(*Itga6 + Itgb1*) and *Mdk*–(*Itga4 + Itgb1*) in mouse FL. Moreover, these identified ligand–receptor pairs can be cross-validated in the omics module of HemAtlas (Figure 6F). For instance, we identified the co-location of the ligand gene *Mdk* expressed in fibroblasts and the receptor gene *Itgb1* expressed in HSPCs in the spatial transcriptomics data from mouse FL (Figure 6F). The detailed process of the cross-species analyses of fibroblasts between zebrafish CHT and mammalian FL is provided in the FAQ section on the HemAtlas website (https://ngdc.cncb.ac.cn/hematlas/faq).

In summary, using various modules for cross-species analyses, we identified two novel conserved marker genes for fibroblasts across three species and elucidated their role in regulating HSPC development through extrinsic cell–cell communication. Our analyses underscore the importance of cross-species conservation in understanding developmental processes, even in hematopoietic organs with distinct architectures. However, the conserved mechanisms by which zebrafish CHT and mammalian FL support HSPC development, despite their architectural differences, require further investigation. More importantly, HemAtlas provides researchers with the tools to perform cross-stage and cross-species analyses for other species, stages, cell types, and genes of interest (https://ngdc.cncb.ac.cn/hematlas/featured/species).

#### Cross-model analyses

This function provides potential insights for engineering the human hematopoietic system *in vitro*, allowing researchers to interactively compare uploaded *in vitro* HSPC scRNA-seq data with available human *in vivo* omics data. Cross-model analyses employ the integrative strategy and involve two steps (Figure 4). The first step is to utilize the SingleCellNet algorithm [40] to assess the degree of HSPC similarity between *in vitro* and *in vivo* based on the pre-built HSPC cross-stage atlas and trained human HSPC classifiers (Figure S6; File S1). The second step of cross-model analyses aims to identify differential key factors of HSPCs across models, including DEGs, enriched GO terms for DEGs, differentially expressed TFs, and differentially expressed cell “differentiation” (CD) surface antigens for cell sorting (see our demo results in https://ngdc.cncb.ac.cn/hematlas/featured/invitro/). These factors may signify differences in the HSPC state between *in vitro* and *in vivo* conditions, offering insights to optimize users’ *in vitro* culture systems. For instance, overexpressing TFs with low expression *in vitro* or sorting more functional HSPCs *in vitro* by CD surface markers could be the potential approaches.

The success of cross-model analyses hinges on the reliable performance of trained human HSPC classifiers. To evaluate the HSPC classifier, after constructed the human HSPC cross-stage atlas (Figure S6A and B; File S1), precision–recall curves were examined in human training data, revealing strong performance during the training of human HSPC classifiers with the specified parameters (Figure S6C). The resulting scores from cell type classification illustrated that the trained HSPC classifiers effectively grouped test HSPCs based on their origin stage labels, while the control group (random) also exhibited self-grouping (Figure S6D). These findings indicate the robust performance of human HSPC classifiers. Additionally, evaluation metrics, such as identification of stage-specific top-ranked gene pairs and the fraction of assigned cell types, demonstrated the effectiveness of the obtained cell classifiers in humans (Figure S6E and F). Leveraging these robust HSPC classifiers, users can upload their *in vitro* HSPC scRNA-seq data to perform cross-model analyses and identify similarity and difference of HSPCs between *in vitro* and *in vivo*. The detailed process of the cross-model analyses is provided in the FAQ section on the HemAtlas website (https://ngdc.cncb.ac.cn/hematlas/faq).

### A comprehensive understanding of HSPC development

**Figure 7:**
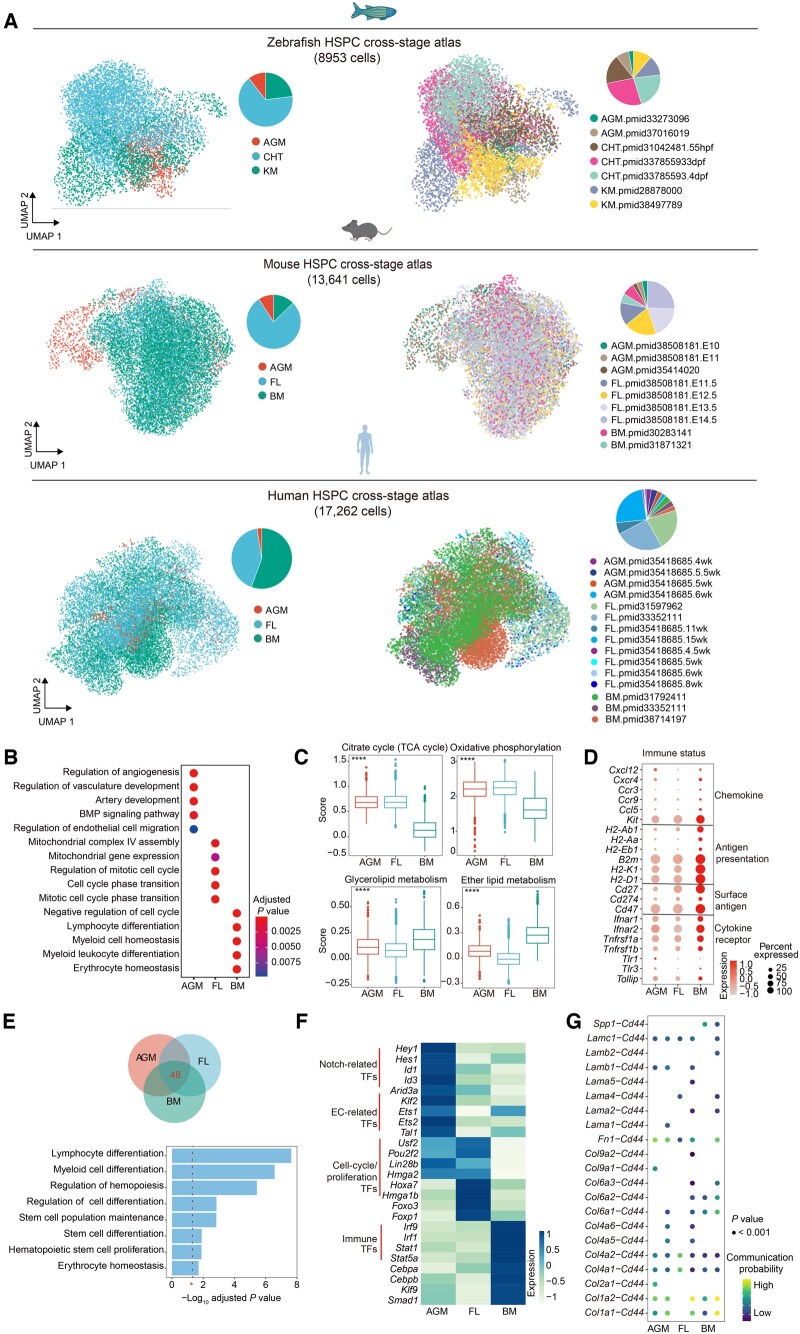
HSPC development module in HemAtlas **A.** Visualization of the HSPC cross-stage atlas for each species. Cells are color-coded by their corresponding cell stages (left) and data sources (right), respectively. The pie plots showing the cell fraction of HSPCs in different developmental stages and data sources for each species. **B**. Dot plot showing GO terms enriched in HSPC-stage differentially expressed genes in each mouse developmental stage. The color of dot represents the adjusted *P* value. **C**. The HSPC metabolic score of representative carbohydrate metabolism and lipid metabolism pathways in three mouse developmental stages. *P* values were determined by Kruskal–Wallis test (****, *P* < 0.0001). **D**. Dot plot showing the stage differentially expressed immune-related genes in mouse HSPCs. The size of the dot corresponds to the percentage of cells expressing the gene in each stage. The color represents the average expression level. The immune-related genes were obtained from a recent study (pmid35418685) [11]. **E**. Mouse 48 stage-common and HSPC-specific TFs and their enriched GO terms. The HSPC-specific and stage-common core TFs were the intersection of HSPC specifically expressed TFs across three hematopoietic stages. The dashed red line corresponds to adjusted *P* value = 0.05. **F**. Heatmap showing stage differentially expressed TFs of mouse HSPCs in three developmental stages. The representative TFs are labeled on left side. The color represents the scaled averaged gene expression level. **G**. Dot plot showing the *Cd44* based ligand–receptor pairs between HSPCs and their dominant regulators (the cell types with the top 2 highest numbers of interactions with HSPCs) across stages. The color of the dot corresponds to the probability of cell–cell communication; the size of the dot represents the *P* value of cell–cell communication. pmid, PubMed identifier number.

HSPCs serve as the seeds of blood, possessing self-renewal ability and multi-lineage differentiation capacity [1]. A systematic understanding of HSPC cross-stage development across species holds the potential to facilitate the acquisition of HSPCs with specific stage features *in vitro*. In the HSPC development module of HemAtlas, we integrated various analysis tools, such as CellOracle [41], CellChat [42], and scMetabolism [43], to comprehensively unveil HSPC cross-stage development for each species at single-cell resolution. At present, the module dedicated to HSPC development has established a comprehensive HSPC cross-stage atlas for each species (**Figure 7**A, Figure S7A–E), and detailed analyses (File S1) reveal the different HSPC subclusters (https://ngdc.cncb.ac.cn/hematlas/hspc/subcluster/) (Figure S7F–H), extensive HSPC inter-stage heterogeneity (https://ngdc.cncb.ac.cn/hematlas/hspc/heterogeneity/) (Figure 7B–D), intrinsic regulation (https://ngdc.cncb.ac.cn/hematlas/hspc/intrinsic) (Figure 7E and F), and extrinsic regulation (https://ngdc.cncb.ac.cn/hematlas/hspc/extrinsic) (Figure 7G), across species.

Users can delve into these segments to investigate stage-specific HSPC cell features and regulation, such as distinct composition of various HSPC subclusters, different HSPC cell metabolic states in different stages, and stage-common or stage-specific key TFs exhibiting high network centrality and expression. Additionally, users can explore important HSPC stage-specific niche regulators (such as fibroblasts) and their corresponding ligand–receptor pairs, providing valuable insights to reveal HSPC stage-specific features for each species.

## Summary and future directions

While some blood databases have been developed using collected scRNA-seq datasets, a comprehensive multi-omics hematopoietic database focusing on cross-stage developmental hematopoiesis across species and human HSPC generation *in vitro* is still lacking. In this study, we introduce HemAtlas, a comprehensive hematopoietic database that integrates multi-omics data derived from both *in vitro* and *in vivo* hematopoietic studies. In comparison with other blood databases, HemAtlas, with its multi-omics data visualization, organ-wide hematopoietic references, three featured functions, and HSPC development module, serves as a valuable resource enabling researchers to investigate hematopoiesis comprehensively in a multi-dimensional and systematic manner. Additionally, we have made all processed data available on the HemAtlas website, which can be accessed at https://ngdc.cncb.ac.cn/hematlas/download.

Continuous efforts are underway to enhance HemAtlas. First, due to potential bias in the selection of scRNA-seq datasets, HemAtlas currently provides a transcriptomic reference for cross-stage developmental hematopoiesis across species. In future updates, additional omics data related to hematopoietic development, such as epigenetics and emerging omics technologies like spatial proteomics [44,45], will be incorporated. This will enable the construction of multi-dimensional hematopoietic references, including a dedicated reference for regulatory elements based on epigenetic data. Moreover, a more comprehensive data integration across stages, species, and omics will provide deeper, more holistic, and unbiased insights into hematopoiesis using advanced scalable integration algorithms. Second, the current version of HemAtlas primarily focuses on cell states rather than cell fates [46]. With the advancement of analytical algorithms based on diverse mathematical models to infer cell developmental trajectories from single-cell data [47,48], alongside the emergence of precise single-cell lineage tracing technologies in hematopoiesis [49–52], the next version of HemAtlas will incorporate insights from these cell fate analyses. Additionally, we will feature an online visualization and exportation interface for constructing phylogenetic trees, enabling users to trace the origins of HSPCs, their downstream differentiation routes, and key driver genes across stages and species. Third, in our HSPC development module, we primarily focus on the inter-stage heterogeneity of HSPCs. However, the inherent intra-stage heterogeneity of HSPCs at each developmental stage requires more systematic exploration. Such an analysis could provide deeper insights into the various HSPC states across different stages and species.

## Supplementary Material

qzaf026_Supplementary_Data

## Data Availability

HemAtlas can be accessed at https://ngdc.cncb.ac.cn/hematlas/. Users can access data and function without the need for registration. HemAtlas has been submitted to Database Commons [53] at the National Genomics Data Center (NGDC), China National Center for Bioinformation (CNCB), which is publicly accessible at https://ngdc.cncb.ac.cn/databasecommons/database/id/8169.

## CRediT author statement

**Zhixin Kang:** Methodology, Software, Investigation, Data curation, Writing – original draft, Writing – review & editing. **Tongtong Zhu:** Investigation, Data curation, Writing – review & editing. **Dong Zou:** Data curation. **Mengyao Liu:** Data curation. **Yifan Zhang:** Writing – review & editing. **Lu Wang:** Conceptualization, Writing – review & editing. **Zhang Zhang:** Conceptualization, Writing – review & editing, Supervision, Project administration, Funding acquisition. **Feng Liu:** Conceptualization, Writing – review & editing, Supervision, Project administration, Funding acquisition. All authors have read and approved the final manuscript.
