# Supplementary material for: HemAtlas: A Multi-omics Hematopoiesis Database
Source: Genomics Proteomics Bioinformatics. 2025 Mar 19;23(2):qzaf026. doi: 10.1093/gpbjnl/qzaf026 (PMC12374576; doi:10.1093/gpbjnl/qzaf026)
Supplement: qzaf026_Supplementary_Data [file qzaf026_supplementary_data.zip › File_S1.docx]

## File S1 Supplementary method

### Data collection and curation

Our criteria for data inclusion require the acquisition of multi-omics data from studies that are directly relevant to the developmental hematopoiesis within classical hematopoietic sites of frequently utilized model organisms (zebrafish, mice, and humans) and hematopoietic stem and progenitor cell (HSPC) *in vitro* culture systems. Specifically, we conducted a systematic search for public omics-related studies on PubMed (<https://pubmed.ncbi.nlm.nih.gov/)> using keywords related to organ/system [aorta–gonad–mesonephros (AGM), caudal hematopoietic tissue (CHT), kidney marrow (KM), fetal liver (FL), bone marrow (BM), direct differentiation, and direct conversion], species/model (humans, mice, zebrafish, and HSPC *in vitro* induction systems), and hematopoiesis (*e.g.*, searching AGM & mice & hematopoiesis for multi-omics data from studies directly relevant to mouse AGM hematopoiesis). The acquired literature for each organ/system was then manually reviewed to categorize the types of omics, including bulk transcriptomic, single-cell transcriptomic (10X Genomics, Smart-seq2, Indrops), spatial transcriptomics (geographical position sequencing (Geo-seq), laser-capture microdissection sequencing (LCM-seq), 10X Visium), and epigenomics [including transposase-accessible chromatin with sequencing (ATAC-seq) and chromatin immunoprecipitation assays with sequencing (ChIP-seq)] (Table S1).

We prioritized retaining the original data analysis results, such as the gene expression matrix and cell annotations, for several reasons. Variations in factors such as age, cell sorting strategies, and sequencing methods across different studies can impact data quality. Consequently, researchers may adopt tailored analysis strategies in individual studies. These strategies might include decisions on whether to remove doublets, the application of different quality control metrics, or the selection of data integration methods to achieve reliable and insightful results. Applying a uniform approach to handling raw data for all collected studies risks losing the original findings of each research piece. This is particularly evident when inconsistencies arise between original results and reanalysis outcomes, as observed in the ABC portal [1].

However, if the original data analysis results were unavailable, standard data analysis was performed to obtain corresponding results. See “Data normalization of single-cell RNA sequencing datasets” and “Data normalization of other omics datasets” for the standard data analysis pipeline for different omics data types.

Additional metadata, such as species, organs, sequencing strategies, cell numbers, data quality metrics, and cell sorting strategies, were also collected (Table S1). To facilitate comparison across multiple datasets, we standardized cell type names using Cell Taxonomy [2] and then manually annotated them. For genes, we simultaneously obtained the correspondence between gene symbols and gene ensemble IDs based on Ensembl and gene annotation based on National Center of Biotechnology Information (NCBI) (https://www.ncbi.nlm.nih.gov/gene/). All omics data retained the original genome version. All the collected datasets can be accessible at <https://ngdc.cncb.ac.cn/hematlas/datasets/>.

### A summary of all the collected publications

HemAtlas currently integrates 94 multi-omics datasets, including 12 organ-wide hematopoietic reference datasets, derived from 43 publications that meet our data inclusion criteria [3–43]. Detailed information can be found at <https://ngdc.cncb.ac.cn/hematlas/publications>. Additionally, we will consistently compile hematopoiesis-related multi-omics data that meets our criteria on a monthly basis.

### Data normalization of single-cell RNA sequencing datasets

Each single-cell RNA sequencing (scRNA-seq) dataset with accessible analysis results, particularly cell annotations, was normalized using the well-established SCTransform method in Seurat (version 3.2.1) [44]. This method employs regularized negative binomial regression to model technical noise in the scRNA-seq data.

When the original analysis results, especially cell type annotations, were unavailable, we processed the scRNA-seq data starting from the raw data. We first attempted to download the gene expression matrix. If the gene expression matrix was not accessible, we downloaded the raw Fast Quality (FASTQ) files from the study links and processed these files using Cell Ranger (with a version aligned to the original studies) with default parameters to generate the expression matrix, ensuring the reference genome version matched those used in the original studies (Table S1). After obtaining the gene expression matrix, we used Seurat (version 3.2.1) for additional data quality control, following the parameters specified in each study (Table S1).

We then employed the SCTransform (version 0.3.3) method to normalize the gene expression matrix, as described above. By identifying 3000 high-variance genes (HVGs), we conducted linear dimension reduction through principal component analysis (PCA) using Seurat’s RunPCA function. The analysis proceeded with non-linear dimension reduction via Uniform Manifold Approximation and Projection (UMAP), using the top 30 principal components to create the cell embedding. Following this, cell clustering was executed with Seurat’s FindNeighbors and FindClusters functions, adjusting the resolution parameter to closely match the cluster numbers reported in the original studies. We identified differentially expressed genes (DEGs) for each cluster using the FindAllMarkers function. Cell type annotations were initially carried out using the Cell Taxonomy database [2], followed by a manual review that incorporated the original study findings and current knowledge.

### Data normalization of other omics datasets

Specifically, for bulk RNA sequencing (RNA-seq) datasets, if both Fragments Per Kilobase of transcript per Million mapped reads (FPKM) (or Reads Per Kilobase per Million mapped reads (RPKM)) matrices and raw count matrices were available, we retained these results or used the countToFPKM (version 1.0) method to convert raw count matrices to FPKM values. Additionally, when only raw sequencing files (FASTQ files) were available, we first assessed their quality using FastQC tools (version 0.11.5). High-quality sequences that passed this evaluation were then aligned to the reference genome using HISAT2 (version 2.1.0) [45], with the reference genome version consistent with that used in each original study (Table S1). Next, SAMtools (version 1.5) was used to convert Binary Alignment Map (BAM) files to Sequence Alignment Map (SAM) format and extract uniquely mapped reads [46]. Finally, the HTSeq tool (version 0.11.2) was employed to count the uniquely mapped reads in each sample using default parameter [47]. For microarray data, the raw outcomes were retained. Cell type and gene symbol standardization were performed as described above.

For each single-cell ATAC-seq (scATAC-seq) dataset, we aligned the raw FASTQ files (or BAM files, if available) to the reference genome (using the original version from the source study) and quantified the data using Cell Ranger ATAC software (version 2.0.0) (Table S1). We then performed scATAC-seq clustering analysis and visualization with ArchR software (version 1.0.1) [48]. Cell annotations were initially obtained from the original study; if these were not available, we cross-referenced with the reference single-cell RNA data to identify cell types. SAMtools was used to derive BAM files from FASTQ files, and bamCoverage in deepTools was employed to convert BAM files to normalized bigWig format [49].

For bulk ATAC-seq or ChIP-seq datasets, SAMtools and bamCoverage were utilized to produce normalized bigWig files. For scATAC-seq datasets, we used the addDeviationsMatrix function in ArchR to compute transcription factor (TF) activity for each TF [48]. The results are available in the “Omics” modules at <https://ngdc.cncb.ac.cn/hematlas/omics>.

### HemAtlas unique IDs

We assigned unique HemAtlas IDs to the gathered hematopoiesis-associated datasets (<https://ngdc.cncb.ac.cn/hematlas/datasets>). To denote the data type, we introduced specific prefixes: “HemAT” signifies ATAC-seq datasets, “HemBK” designates bulk RNA-seq datasets, “HemCP” represents ChIP-seq datasets, “HemSC” stands for scRNA-seq datasets, and “HemST” is used for spatial transcriptome datasets.

### Construction of organ-wide hematopoietic references

*Criteria for selecting data*

As illustrated in the data inclusion criteria mentioned above, the multi-omics data that we have gathered come from canonical hematopoietic sites in commonly used model animals, with a focus on HSPC cross-stage development. Consequently, many of the collected datasets are enriched for HSPCs. For example, the scRNA-seq dataset HemSC00000011 includes sorted Lineage-Sca-1^+^cKit^+^ (LSK) and Lineage^-^ cKit^+^ (LK) cells from mouse BM, which are rich in hematopoietic stem cells (HSCs) and hematopoietic progenitor cells (HPCs) [36]. Additionally, other scRNA-seq datasets, such as those from mouse FL and zebrafish CHT, are also highly enriched for HSPCs [9,32].

Therefore, in constructing the organ-wide hematopoietic references, we prioritized datasets with a variety of data types to ensure a comprehensive representation of niche cell types. For instance, we excluded the scRNA-seq dataset HemSC00000011 when creating the mouse BM atlas and the mouse cross-stage reference. The scRNA-seq data used for the construction of organ-wide hematopoietic references including: zebrafish AGM: PMID: 37016019 [11] and PMID: 33273096 [27]; zebrafish CHT: PMID: 33785593 [12] (including three time points: 55 h post-fertilization, 3 days post-fertilization, and 4 days post-fertilization) and PMID: [31042481](https://www.ncbi.nlm.nih.gov/pubmed/31042481) [9]; zebrafish KM: PMID: 38497789 [29] and PMID: 28878000 [14]; mouse AGM: PMID: 35414020 [22] and PMID: 38508181 [6] [including two time points: embryonic day (E10) and E11]; mouse FL: PMID: 34341490 [32] (including four time points: E11.5, E12.5, E13.5, and E14.5); mouse BM: PMID: 31871321 [42] and PMID: 30283141 [43]; human AGM: PMID: 35418685 [40] (including four time points: 4 weeks, 5 weeks, 5.5 weeks, and 6 weeks); human FL: PMID: 31597962 [20], PMID: 33352111 [19], and PMID: 35418685 [40]; human BM: PMID: 33352111 [19], PMID: 38714197 [41], and PMID: 31792411 [50] (Table S1).

*Data integration*

To construct organ-wide hematopoietic references for each species, for the selected datasets mentioned above, we downloaded their corresponding raw FASTQ files from Sequence Read Archive (SRA) database (<https://www.ncbi.nlm.nih.gov/sra>), Genome Sequence Archive (GSA) database (<https://ngdc.cncb.ac.cn/gsa>/), or ArrayExpress database (<https://www.ebi.ac.uk/biostudies/arrayexpress>). Following quality control of the raw FASTQ files using FastQC tools (version 0.11.5), high-quality sequences were aligned to the reference genome with Cell Ranger (version 7.1.0) with default parameters. The reference genomes used were GRCz11 for zebrafish, mm10 for mice, and GRCh38 for humans (Table S1). Next, for the cell-by-gene expression matrix obtained from each dataset, we calculated standard quality control metrics, including the number of unique genes detected per cell, the total number of molecules detected per cell, and the percentage of reads mapping to the mitochondrial genome. Then for each dataset, an observation was uniformly defined as an outlier if it deviated by more than a specified number of median absolute deviations (MADs), with the default set to 3, from the median in these quality control metrics. After removing these outliers, each dataset was used to construct a corresponding Seurat object (version 3.2.1), and the well-established SCTransform method in Seurat was applied to normalize the raw expression matrix data and we obtained 3000 HVGs for each dataset through SCTransform normalization.

For datasets from each stage within each species, we began by integrating data from individual stages to create stage-specific atlases. The PrepSCTIntegration function was used to calculate informative HVGs across datasets for each stage. These HVGs were then utilized to identify integration anchors with the FindIntegrationAnchors function, and the data was integrated using the IntegrateData function. PCA was subsequently performed with the RunPCA function, and the top 30 principal components were selected for UMAP analysis and visualization using the RunUMAP function. It’s important to note that the canonical correlation analyses (CCA)-corrected gene expression matrix was only used for visualization-related analyses. It should be noted that some datasets include multiple time points, such as the mouse FL dataset (PMID: 34341490), which comprises four time points: E11.5, E12.5, E13.5, and E14.5. Data from each time point within these datasets were processed as described above.

Next, the Louvain algorithm was employed for cell clustering using the FindNeighbors and FindClusters functions in Seurat, based on the top 30 principal components. DEGs were then identified using the RunPrestoAll function on the SCTransform-normalized expression matrix. Initial cell type annotations were carried out using the Cell Taxonomy database, followed by manual review that incorporated the original study findings and current knowledge. The markers used for cell cluster annotation are listed in Table S3.

After constructing stage-specific atlases for each species, we integrated all three stage-specific atlases (*i.e.*, AGM, CHT/FL, and KM/BM) for each species to create corresponding cross-stage references, as described above. It is important to note that the cell annotation of the cross-stage references was based on the original stage-specific atlases for each stage in each species to ensure more accurate cell annotation.

*The comparison of different integration methods*

To make sure an appropriate data integration method for constructing the cross-stage single-cell atlas reference, we evaluated commonly used methods, including mutual nearest neighbors (MNN) (version 1.2.4) and Harmony (version 0.1.0) during the construction of mouse cross-stage reference [51,52]. First, the integration of datasets from different stages using MNN and Harmony was performed with the default workflow, employing the RunFastMNN and RunHarmony functions in Seurat (version 3.2.1) on the SCTransform-normalized expression matrix. Then, we employed the local inverse Simpson’s index (LISI) method for a quantitative assessment of data integration performance [52]. This analysis was conducted using the lisi::compute_lisi function with default parameters and the statistical comparison of different data integration methods, with no integration group, was performed using the *t*-test method.

Subsequently, we detected the expression of cell-type-specific markers, such as *Hlf* (indicative of HSPCs) and *Pou5f1* (indicative of germ cells), to demonstrate whether the same cell types were clustered together across different stages. Specifically, we firstly observed that method CCA exhibited a superior integration capability compared to other commonly used methods in mice normalized datasets (Figure S3A and B). Furthermore, CCA demonstrated the ability to successfully coalesce *Hba-a1*-positive erythrocyte and *Pou5f1*-positive germ cell separately (Figure 3G). We then examined the expression of the HSPC-specific marker *Hlf* across various integration methods and found only the CCA method successfully clustered *Hlf*-positive cells together (Figure 3G, Figure S3C).

Next, to further examine potential batch effects after CCA correction in the mouse cross-stage reference, we performed the k-nearest neighbor batch effect test (kBET) analysis both before and after data correction using the kBET function from the R package kBET (version 0.99.6) [53] with the default parameters. Statistical significance between the expected and observed rejection rates before and after data correction, as well as between the observed rejection rates before and after correction, was assessed using the *t*-test*.*

*The cross-species integration*

It is important to note that we did not perform systematic cross-species integration due to the potential loss of valuable biological information. For example, certain zebrafish genes, such as *fli1a* and *fli1b*, which are one-to-many homologous genes, may be obscured after homologous gene conversion, where they are merged into a single category, *FLI1*. This can obscure the distinct expression patterns of *fli1a* and *fli1b* observed in zebrafish KM (<https://ngdc.cncb.ac.cn/hematlas/dataset/HemSC00000043>). Similarly, cross-species integration can mask the cross-stage dynamics of other paralogous genes, such as *csf1rb*.

### Construction of cross-stage and cross-species analysis

*A summary of two strategies for cross-dataset comparison*

For each individual collected scRNA-seq dataset (where we prioritized retaining the original data analysis results such as cell type annotation) and each constructed stage-specific atlases of three species (where cell type annotations were systematically re-annotated manually), we employed various analysis modules — such as cell overview, gene expression, cell components, DEGs (pre-calculated for each dataset), Gene Ontology (GO) enrichment (pre-calculated for each dataset), and cell–cell interactions (pre-calculated for each dataset) — to facilitate cross-dataset comparisons (Figure 4). These modules showcased the individual outcomes of each dataset using a non-integrative strategy.

Moreover, we constructed cross-stage references for each species from raw FASTQ files using a uniform analysis workflow for each species that enable direct cross-stage comparisons through an integrative strategy. We did not perform systematic cross-species integration, as discussed above in “The cross-species integration”.

By carefully balancing the preservation of original analysis results with the need for thorough and uniform data integration, we developed two strategies for cross-dataset analysis: the non-integrative strategy and the integrative strategy.

Non-integrative strategy

For cross-stage analysis, researchers can explore individual scRNA-seq datasets online, which preserve the original analysis results of each dataset, alongside stage-specific atlases that we constructed with systematic and manual cell annotations. This allows for cross-dataset comparisons without the need for data integration.

For cross-species analysis, to avoid potential loss of information due to homologous gene conversion, we did not perform cross-species integration. Instead, researchers can use individual scRNA-seq datasets alongside stage-specific atlases that we constructed to perform cross-dataset comparisons without the need for cross-species integration.

Integrative strategy

For cross-stage analysis, researchers can utilize constructed cross-stage references for each species, which involve systematic data integration and manual cell annotations, to perform direct cross-dataset comparisons. This allows for the analysis of cell fraction and gene expression dynamics across different developmental stages, providing insights into the temporal progression of cellular and molecular changes.

Taken together, these approaches (non-integrative and integrative strategy) are designed to enhance the utility and reliability of our resource for the research community, fostering deeper insights into hematopoiesis across various stages and species. Additionally, two supplementary case studies were provided to demonstrate the application of these two strategies for cross-dataset analysis (Figures 5 and 6) (<https://ngdc.cncb.ac.cn/hematlas/faq>).

*Non-integrative strategy*

Differentially expression analysis

Utilizing SCTransform-normalized data, we employed the FindAllMarkers function from the Seurat package (version 3.2.1) to discern cell type-specific DEGs by compare each cell types to all other cell types in each dataset (individual collected scRNA-seq dataset and constructed stage-specific atlases of three species). For example, we compared the fibroblast with other cell types in each selected dataset to calculate fibroblast-specific DEGs in that dataset. *P* value adjustment was carried out through Bonferroni correction based on the total number of genes in the dataset. Specific parameters were set, including thresh.use at 0.25, min.pct at 0.1, and only.pos as TRUE. The resulting cell type or sub-cell cluster specific DEGs associated within each dataset were visualized in the “DEGs” module within HemAtlas.

GO enrichment analysis

The clusterProfiler package (version 3.14.3) was employed to enrich the GO pathways of the gene lists of interest for each species [54]. The parameters “pvalueCutoff” and “qvalueCutoff” were set at 0.05 and 0.2, respectively. Concurrently, GO terms enriched in cell type-specific DEGs for each dataset were visualized in the “GO enrichment” module of HemAtlas.

Ligand–receptor network

CellChat (version 1.6.1) was employed to infer the cell–cell communication network across organs and species (only for datasets with niche cells) [55]. Initially, SCTransform-normalized data was utilized to construct a CellChat object. Subsequently, we selected all ligand–receptor interaction databases, including Secreted Signaling, ECM-Receptor, and Cell-Cell Contact, to compute the communication probability and infer the cellular communication network using the computeCommunProb function in CellChat. It’s worth noting that the cell sorting strategies applied in each collected scRNA-seq dataset could impact the cell–cell communication analysis, as specific enrichment strategies might lead to the omission of some rare niche cells. However, regarding the impact of changes in cell numbers due to cell sorting strategies on cell–cell communication strength, the CellChat analysis accounts for these variations by removing the cell number effect when calculating communication strength between HSPCs and niche cells (such as endothelial cells and fibroblasts) [55].

*Integrative strategy*

Similar to the “Omics” module ([https://ngdc.cncb.ac.cn/hematlas/omic](https://ngdc.cncb.ac.cn/hematlas/omics)[s](file:///C:\Users\kzx\AppData\Roaming\Microsoft\Word\s)) that we developed in HemAtlas, we also constructed the organ-wide “Hematopoietic References” module in HemAtlas (<https://ngdc.cncb.ac.cn/hematlas/references>). This module allows researchers to interactively explore and visualize constructed stage-specific atlases and cross-stage references online based on their scientific interests.

Furthermore, in the cross-stage and cross-species analysis modules, we have provided corresponding constructed stage-specific atlases datasets, enabling users to perform cross-dataset analysis with more uniform cell annotation using these stage-specific atlases. Additionally, in the cross-stage analysis module, we offer an analysis selection of the integrative strategy, allowing users to jump to the corresponding cross-stage references for direct cross-dataset comparisons. By selecting the intersected cross-stage reference, researchers can directly perform cross-stage analyses, such as comparing gene expression and cell composition across different stages.

### Construction of cross-model analysis

*Training of cell classifiers based on in vivo data*

Before users upload *in vitro* HSPC scRNA-seq data, we employed the SingleCellNet package (version 0.1.0) to train cell classifiers based on our constructed human HSPC cross-stage atlases (Construction of HSPC cross-stage atlases) [56]. To enhance classifier robustness and mitigate noise, we firstly calculated 3000 HVGs for human HSPC integrated scRNA-seq data. Subsequently, the scn_train function in SingleCellNet was utilized to train the cell classifiers. Considering the varying cell numbers of HSPCs at different stages, the number of control random groups was set between 50–200. The scn_predict function was then employed to assess the performance of cell classifiers. After evaluating the precision–recall curve, classification score, and cell attribution, we determined that setting the number of control random groups to 100 yielded optimal performance for human *in vivo* HSPCs scRNA-seq data.

*Identification of similarity between HSPCs in vivo and in vitro*

After identifying cell classifiers with good performance, users uploading *in vitro* HSPC scRNA-seq data will undergo normalization using SCTransform mentioned above. Subsequently, we will map onto the *in vivo* HSPC atlas based on the trained classifiers using 3000 HVGs. The number of control random groups for data mapping equals the *in vitro* HSPC cell number, and the scn_predict function will be utilized to calculate classification scores. The resulting data will be visualized through heat maps or violin plots.

*Identification of differences between HSPCs in vivo and in vitro*

The *in vivo* HSPC transcriptome data with the highest classification scores and the *in vitro* data were integrated using the CCA algorithm as mentioned above. Subsequently, DEGs, differentially expressed TFs, surface cell “differentiation” (CD) surface antigens, and DEG-enriched GO terms between HSPCs *in vivo* and *in vitro* were computed, as described above.

### Construction of HSPC development module

*Construction of HSPC cross-stage atlases*

Based on the constructed cross-stage references mentioned above, we focused on the HSPCs across stages for each species and selected them to construct the HSPC cross-stage atlas. The PCA embeddings were obtained from the corresponding cross-stage references, as these CCA-adjusted PCA embeddings perform well during cross-stage integration. We then re-performed UMAP analysis and visualization using the RunUMAP function for thses HSPC cross-stage atlases.

It’s noteworthy that we did not perform cross-species data integration, mainly due to that the commonly used cross-species integration methods may inadvertently overlook non-homologous genes information as mentioned above. In our attempts, we identified only 12,301 homologous genes among the three species based on homologene R package (version 1.4.68) (Figure S7E). However, the individual HSPC atlases for zebrafish, mouse, and human contain 28,581, 26,773, and 38,696 detected genes, respectively, which implies that homologous gene conversion results in some loss of information. Meanwhile, in the identification of key informative TFs, we found some zebrafish genes (one-to-many homologous genes), such as *fli1a* and *fli1b*, could not be detected. Given that certain species-specific non-homologous genes may also play a significant role in the regulation of hematopoiesis [11,57], we did not perform cross-species integration in HSPC module.

*Validation of constructed HSPC cross-stage atlases*

Similar to the validation of the constructed cross-stage references mentioned above, to validate the constructed HSPC cross-stage atlases, we first performed kBET analysis both before and after CCA data correction using the kBET function from the R package kBET (version 0.99.6) in mouse HSPCs. Statistical significance between the expected and observed rejection rates before and after data correction, as well as between the observed rejection rates before and after correction, was assessed using the *t*-test method.

Next, we evaluated commonly used methods, including MNN (version 1.2.4) and Harmony (version 0.1.0), during the construction of the mouse HSPC cross-stage atlases. Similarly, we employed the LISI method for a quantitative assessment of data integration performance. This analysis was conducted using the lisi::compute_lisi function with default parameters, and statistical comparisons of different data integration methods, including the no-integration group, were performed using the *t*-test method.

*Identification of HSPC subclusters across stages and species*

To achieve a more precise identification of HSPC subclusters across different stages for each species, we performed differential gene expression analysis using the constructed cross-stage HSPC atlases in a stage-specific manner. Specifically, for each stage in each species, HSPCs were re-clustered using the FindNeighbors and FindClusters functions in Seurat (version 3.2.1). DEGs were identified using the RunPrestoAll function on the SCTransform-normalized expression matrix. The clustering resolution parameter was set to 1 to enable the identification of sufficient subclusters. To assess the purity of the identified HSPC subclusters, the R package ROGUE (version 1.0) was employed [58], with the threshold set at 0.85. Subsequently, these subclusters were manually re-annotated based on well-established marker genes to refine the classification of HSPC subclusters. Specifically, the subclusters with significantly higher expression of lineage markers (such as *HBG*, *LYZ*, and *CD79A*) were identified as lineage-biased HSPCs. Subclusters with significantly higher expression of endothelial cell markers (such as *GJA5* and *CDH5*) were identified as nascent HSPCs. Subclusters with significantly higher expression of proliferating markers (such as *MKI67* and *TOP2A*) were identified as proliferating HSPCs. Similarly, subclusters with significantly higher expression of stem cell markers (such as *MYB* and *HLF*) were identified as stem HSPCs, and subclusters with significantly higher expression of quiescence-related markers (such as *Ly6a* and *FOXO3*) were identified as quiescent HSPCs.

*Identification of HSPC inter-stage heterogeneity*

Differentially expression and GO enrichment analysis

The differentially expression analysis was performed as described above to identify DEGs of HSPCs between different stages for each species. The stage-specific DEGs were then used to perform GO enrichment analysis using the clusterProfiler package as described above.

Calculation of metabolic activity

Following the guidelines [59], we computed the metabolic activity of HSPCs at different developmental stages using the constructed HSPC cross-stage atlas for each species. Utilizing the Kyoto Encyclopedia of Genes and Genomes (KEGG) database, we employed the “AUCell” method to calculate the metabolic activity of each cell across 85 KEGG metabolism pathways, using the “sc.metabolism.Seurat” function in scMetabolism (version 0.2.1). Mouse and zebrafish data were first homologously transferred into human gene names. Subsequently, the same analysis was performed as in the human dataset.

Cell cycle score

We employed the “CellCycleScoring” function in Seurat to assign each cell a cell cycle score based on its expression of G2/M and S phase markers. The cell cycle marker sets for mice and zebrafish were transformed from human homologous genes.

The visualization of immune and signaling genes

The immune genes were obtained from a recent study [40], while signaling genes were sourced from the KEGG database (https://www.genome.jp/kegg/). We utilized dot plots to visualize the expression profiles of these genes across the three development stages in each species. In the dot plots, color represents the average expression of the genes, and dot size corresponds to the gene expression fraction.

*Identification of intrinsic regulation of HSPCs*

Identification of stage-common and stage-specific TFs for each species

To obtain HSPC-specific features, we performed differential expression analysis for each stage-specific atlas for each species. We compared HSPCs against other cell types to identify HSPC-specific DEGs. These DEGs were considered as markers specific to HSPCs within that particular hematopoietic stage. Subsequently, the intersection of HSPC markers, focusing on TFs, across the three distinct hematopoietic stages for each species was termed as the set of HSPC-specific and stage-common core TFs for that species. Following the removal of stage-common core TFs for each species, we performed differential expression analysis on the constructed HSPC cross-stage atlas to identify HSPC differentially expressed TFs, termed as stage-specific TFs for HSPCs in each species.

Calculation of stage-specific TF network scores of HSPCs

To identify the TFs essential for each developmental stage with high network scores across species, we began by selecting both stage-common and stage-specific TFs, along with 3000 HVGs from each HSPC cross-stage atlas for analysis using CellOracle (version 0.15.0) [60]. Using pre-existing gene regulatory networks (GRNs) for each species, we applied the oracle.get_links function in CellOracle to compute GRNs for the HSPC cross-stage atlas with default settings. We then filtered the network edges, removing those with a *P* value greater than 0.01 and weak connections, and retained the top 2000 significant edges based on edge strength. Following this, we used the links.get_network_score function to calculate network scores for HSPCs at each stage, incorporating various centrality metrics. We specifically ranked TFs based on degree centrality, which quantifies the number of direct connections that a TF has to other TFs in a GRN, with a higher degree indicating a more central role. Additionally, we calculated the TF–target gene networks for these highly informative TFs.

Construction of extrinsic cell–cell communication of HSPCs

Similarly, CellChat (version 1.6.1) was employed to construct extrinsic cell–cell communication of HSPCs as mentioned above for our construed stage-specific atlases. By setting HSPCs as target cell types, the top two interacting cell types with HSPCs were determined as the dominant regulators for each organ. These determinations were based on ligand–receptor interaction pair numbers. Following this, the cell–cell interaction pairs between HSPCs and the niche cells were visualized and presented in the HSPC developmental model.

### Experimental validation

*Fluorescence in situ hybridization*

Fluorescence *in situ* hybridization was used to detect the expression of *sparc* and *igfbp5b* in zebrafish. The protocol was performed similarly to the whole mount in situ hybridization (WISH) before the antibody incubation as previously described [9]. After removing antibody and washing embryos with phosphate buffered saline with 0.1% Tween-20 (PBST), embryos were stained with tyramide signal amplification (TSA)- fluorescein isothiocyanate (FITC) amplification reagent (1:100). Before immunofluorescence staining, the first color reaction was stopped by gradient methanol. After removing the reaction buffer, embryos were washed sequentially by 25%, 50%, and 75% methanol/PBST (10 min/each buffer), 1% H_2_O_2_/methanol (30 min), 75%, 50%, and 25% methanol/PBST (10 min/each buffer), and PBST (2 × 35 min). The sequences of the probe primers used are listed in Table S4.

*Quantitative real-time polymerase chain reaction*

Total RNA of sorted cells was extracted by a QIAGEN RNeasy Mini Kit (Cat. No. 74104; manufacturer: QIAGEN; nationality: German; city: Hilde) and then reversely transcribed by oligo-dT to obtain complementary DNA (cDNA) as quantitative real-time polymerase chain reaction (qPCR) templates. qPCR assays were performed with a Bio-Rad system, and the expression of *Gapdh* for mouse was used as the internal control. The sequences of the qPCR primers used are listed in Table S4.

### Database contents and usage

*HemAtlas overview*

HemAtlas stands as an accessible and user-friendly database resource for hematopoiesis. The diverse omics data compiled from hematopoietic organs/systems involved in HSPC development (AGM, CHT, KM, BM, direct differentiation, and direct conversion) in major research models (human, mice, zebrafish, and HSPC *in vitro* induction) have been normalized, providing an interactive platform for visualization and exploration within the “Omics” module. Moreover, based on scRNA-seq datasets, we constructed the organ-wide hematopoietic references for each species using a unified analysis workflow starting with raw FASTQ files. Furthermore, three featured functions — single-cell cross-stage, cross-species, and cross-model analysis — facilitate a comprehensive understanding of developmental hematopoiesis and the evolution of the hematopoietic system *in vivo*, and also offer potential insights for engineering users’ hematopoietic systems *in vitro.* Of particular significance, the integrated “HSPC Development” module has constructed a comprehensive HSPC cross-stage development encyclopedia, enabling the decoding of HSPC *in vivo* development and guiding the generation of HSPCs *in vitro*. The database comprises various modules to fulfill functions such as homepage, omics, hematopoietic references, knowledge, metadata, featured functions, HSPC development, statistics, help, and download.

*Homepage*

On the homepage, the “Search” facilitates data search based on multiple layers including species, stages, cell types, and genes. The “Publications” section provides information on the latest studies in hematopoiesis. Conveniently, “Featured Functions” serves as a gateway for cross-stage, cross-species, and cross-model analysis. “Data Statistics” dynamically presents current dataset statistics. The “Encyclopedia” section offers a comprehensive review of cross-stage hematopoiesis knowledge in each species and culture system, encompassing descriptions of hematopoietic organs and corresponding major hematologic events.

*Omics*

The “Omics” module offers simultaneous omics visualization for users engaged in multiple omics studies, as mentioned previously. Users can select their preferred datasets from the navigation bar across four different omics. By utilizing the zoom-in button, users can seamlessly transition to a specific dataset, gaining access to detailed information.

*Knowledge*

The “Knowledge” module represents a free encyclopedia of hematopoiesis in different species/models, encompassing human hematopoiesis, mouse hematopoiesis, zebrafish hematopoiesis, and *in vitro* hematopoiesis. Specifically, human hematopoiesis involves organs such as AGM, FL, and BM; mouse hematopoiesis includes AGM, placenta, FL, and BM; zebrafish hematopoiesis covers AGM, CHT, and KM; and *in vitro* hematopoiesis is associated with direct differentiation and direct conversion. In this module, users can access not only datasets but also knowledge information about hematopoiesis.

*Hematopoietic References*

To remove potential batch effects and sequencing inconsistencies across different datasets when constructing organ-wide hematopoietic references, HemAtlas re-analyzes selected scRNA-seq datasets starting from raw FASTQ files using a unified analysis workflow that includes manual re-annotation of cell types. By standardizing the analysis pipeline and ensuring consistent cell type annotation, the “Hematopoietic References” module in HemAtlas provides organ-wide hematopoietic references for each species, which included stage-specific atlases and cross-stage references.

*Metadata*

In the “Metadata” section, users can access to metadata information corresponding to each dataset. First, all datasets and samples are encoded with unique HemAtlas IDs for easy identification. And for cell types, HemAtlas provides both the original sub-cell type names and curated merged cell type names, allowing users to study interested cell types or sub-cell clusters. Additionally, HemAtlas interactively displays the annotation of detected genes in collected datasets, encompassing 94,769 genes [including protein-coding genes, ribosomal RNA (rRNA), noncoding RNA (ncRNA), *etc*.] in humans and *in vitro* systems (37,550 genes), mice (36,577 genes), and zebrafish (20,642 genes). Users can explore the “Genes” section to select items of interest and obtain homologous genes in all three species. All publications associated with datasets in HemAtlas are showcased through the Open Library of Bioscience.

*Featured Functions*

HemAtlas features user-friendly tools designed for demand-driven exploration of hematopoiesis both *in vivo* and *in vitro*. Leveraging individual collected scRNA-seq dataset (where we prioritized retaining the original data analysis results such as cell type annotation) and each constructed organ-wide hematopoietic references of three species (where cell type annotations were systematically re-annotated manually), we have implemented various interactive analysis modules for cross-stage, cross-species, and cross-model analyses function.

*Cross-stage analysis*

Cross-stage analysis aims to reveal development of hematopoiesis in each species. Users can make a custom selection in only one species but 1–4 organs for multi-stages comparison. Cross-stage analysis includes following analysis modules.

Cell overview and gene expression

There are visualizations of cell types, gene expression, and cell distributions across-stages for each selected dataset. Users can explore intersected gene expression or interested cell type distributions. Specifically, for the visualization of gene expression, cells with zero gene expression values are removed from the violin plots to maintain data load efficiency and webpage fluency. Additionally, for some scRNA-seq data containing a large number of cells, we limit the feature plot to display approximately 3000 genes. However, we provide a “Dot plot” section that displays both the average cell expression (avg.exp) and the percentage of cell expression (pct.exp) for all the genes in each dataset.

DEGs

Pre-calculated DEGs for each cell type/sub-cell cluster enables interactive exploration of important genes for each dataset.

GO enrichment

Users can access cell-type-specific or sub-cell-type-specific enriched GO pathways in each dataset. It should be noted that displaying all thousands of GOs in the drop-down box is impractical. Instead, the drop-down box includes a search function, allowing researchers to search by GO ID and GO description.

Cell–cell interaction

Cell–cell interactions, calculated by CellChat, display cell–cell communication strength and underlying ligand–receptor pairs.

Cell components

Cell components help users obtain cell type distribution in different organs.

Meanwhile, in the cross-stage analysis module, HemAtlas provides an integrative strategy for direct cross-dataset comparisons.

*Cross-species analysis*

Cross-species analysis aims to reveal the evolutionary conservation and difference of hematopoiesis. Usage between cross-stage analysis and cross-species analysis is similar for cell overview, gene expression, DEGs, GO enrichment, and cell–cell interaction, and cell components.

*Cross-model analysis*

Real-time analysis compares uploaded HSPC *in vitro* data with the constructed *in vivo* transcriptomic reference for HSPCs, covering various hematopoietic organs for each species. Online analysis results include summaries, similarity assessments, DEGs, GO analyses, differentially expressed TFs, and differentially expressed CD surface markers. All results are available for download in table or figure formats.

*HSPC Development*

In the integrated analysis results for HSPC cross-stage development among three species (human, mice, and zebrafish), HemAtlas offers an interactive exploration HSPC development module in four main sections.

Subcluster analysis

User can interactively explore cell composition of HSPC subclusters and gene expression profiles across various developmental stages for each species.

Inter-stage heterogeneity

Users can interactively explore dynamic gene expression, different GO terms, diverse cell cycle states, cell metabolism, and immune states during HSPC cross-stage development for each species.

Intrinsic regulation

Users can access to HSPC stage-common or stage-specific key TFs for each species based on TF expression and network centrality pre-calculated.

Extrinsic regulation

This section displays stage-specific important HSPC niche regulators and the underlying ligand–receptor pairs interacting with HSPCs across stages and species.

*Statistics*

In the “Statistics” module of HemAtlas, users can access a controlled word cloud covering omics, species, organs, and cell types based on samples in datasets. For cell types, we have constructed a hematopoiesis-associated cell type tree within HemAtlas, linking these cells to the “Cell Taxonomy” database for additional information such as cell ID, cell synonyms, cell type descriptions, cell markers across tissues, similar cells, and more. Additionally, users can obtain statistics for dataset groups and sample groups based on omics, species, and organs.

*Help*

To better illustrate the application of HemAtlas, we have included a detailed description of the online usage of HemAtlas and four case studies in the “FAQ” section on the HemAtlas website to provide users with practical operation instructions and step-by-step guidance. Moreover, in the “Contact Us” section, we have provided our email address so that users can directly contact us if they have any questions.

*Download*

All the processed data, which includes bulk RNA-seq, scRNA-seq, spatial transcriptomic, and epigenomics data along with their corresponding metadata, can be publicly downloaded in an interactive manner. Additionally, the stage-specific atlases, cross-stage references, and HSPC cross-stage atlases across three species are also available for public access.

**References**

[1] Gao X, Hong F, Hu Z, Zhang Z, Lei Y, Li X, et al. ABC portal: a single-cell database and web server for blood cells. Nucleic Acids Res 2023;51:D792–804.

[2] Jiang S, Qian Q, Zhu T, Zong W, Shang Y, Jin T, et al. Cell Taxonomy: a curated repository of cell types with multifaceted characterization. Nucleic Acids Res 2023;51:D853–60.

[3] Zhu Y, Wang T, Gu J, Huang K, Zhang T, Zhang Z, et al. Characterization and generation of human definitive multipotent hematopoietic stem/progenitor cells. Cell Discov 2020;6:89.

[4] Zhu Q, Gao P, Tober J, Bennett L, Chen C, Uzun Y, et al. Developmental trajectory of prehematopoietic stem cell formation from endothelium. Blood 2020;136:845–56.

[5] Zheng Z, Yang S, Gou F, Tang C, Zhang Z, Gu Q, et al. The ATF4–RPS19BP1 axis modulates ribosome biogenesis to promote erythropoiesis. Blood 2024;144:742–56.

[6] Zhang Y, Kang Z, Liu M, Wang L, Liu F. Single-cell omics identifies inflammatory signaling as a trans-differentiation trigger in mouse embryos. Dev Cell 2024;59:961–78.e7.

[7] Zhang P, He Q, Chen D, Liu W, Wang L, Zhang C, et al. G protein-coupled receptor 183 facilitates endothelial-to-hematopoietic transition via Notch1 inhibition. Cell Res 2015;25:1093–107.

[8] Zeng Y, He J, Bai Z, Li Z, Gong Y, Liu C, et al. Tracing the first hematopoietic stem cell generation in human embryo by single-cell RNA sequencing. Cell Res 2019;29:881–94.

[9] Xue Y, Liu D, Cui G, Ding Y, Ai D, Gao S, et al. A 3D atlas of hematopoietic stem and progenitor cell expansion by multi-dimensional RNA-seq analysis. Cell Rep 2019;27:1567–78.e5.

[10] Xie X, Liu M, Zhang Y, Wang B, Zhu C, Wang C, et al. Single-cell transcriptomic landscape of human blood cells. Natl Sci Rev 2021;8:nwaa180.

[11] Xia J, Liu M, Zhu C, Liu S, Ai L, Ma D, et al. Activation of lineage competence in hemogenic endothelium precedes the formation of hematopoietic stem cell heterogeneity. Cell Res 2023;33:448–63.

[12] Xia J, Kang Z, Xue Y, Ding Y, Gao S, Zhang Y, et al. A single-cell resolution developmental atlas of hematopoietic stem and progenitor cell expansion in zebrafish. Proc Natl Acad Sci U S A 2021;118:e2015748118.

[13] Vo LT, Kinney MA, Liu X, Zhang Y, Barragan J, Sousa PM, et al. Regulation of embryonic haematopoietic multipotency by EZH1. Nature 2018;553:506–10.

[14] Tang Q, Iyer S, Lobbardi R, Moore JC, Chen H, Lareau C, et al. Dissecting hematopoietic and renal cell heterogeneity in adult zebrafish at single-cell resolution using RNA sequencing. J Exp Med 2017;214:2875–87.

[15] Sugimura R, Jha DK, Han A, Soria-Valles C, da Rocha EL, Lu YF, et al. Haematopoietic stem and progenitor cells from human pluripotent stem cells. Nature 2017;545:432–8.

[16] Shen J, Xu Y, Zhang S, Lyu S, Huo Y, Zhu Y, et al. Single-cell transcriptome of early hematopoiesis guides arterial endothelial-enhanced functional T cell generation from human PSCs. Sci Adv 2021;7:eabi9787.

[17] Schönberger K, Obier N, Romero-Mulero MC, Cauchy P, Mess J, Pavlovich PV, et al. Multilayer omics analysis reveals a non-classical retinoic acid signaling axis that regulates hematopoietic stem cell identity. Cell Stem Cell 2022;29:131–48.e10.

[18] Rubin SA, Baron CS, Pessoa Rodrigues C, Duran M, Corbin AF, Yang SP, et al. Single-cell analyses reveal early thymic progenitors and pre-B cells in zebrafish. J Exp Med 2022;219:e20220038.

[19] Ranzoni AM, Tangherloni A, Berest I, Riva SG, Myers B, Strzelecka PM, et al. Integrative single-cell RNA-seq and ATAC-seq analysis of human developmental hematopoiesis. Cell Stem Cell 2021;28:472–87.e7.

[20] Popescu DM, Botting RA, Stephenson E, Green K, Webb S, Jardine L, et al. Decoding human fetal liver haematopoiesis. Nature 2019;574:365–71.

[21] McKinney-Freeman S, Cahan P, Li H, Lacadie SA, Huang HT, Curran M, et al. The transcriptional landscape of hematopoietic stem cell ontogeny. Cell Stem Cell 2012;11:701–14.

[22] Lummertz da Rocha E, Kubaczka C, Sugden WW, Najia MA, Jing R, Markel A, et al. CellComm infers cellular crosstalk that drives haematopoietic stem and progenitor cell development. Nat Cell Biol 2022;24:579–89.

[23] Lis R, Karrasch CC, Poulos MG, Kunar B, Redmond D, Duran JGB, et al. Conversion of adult endothelium to immunocompetent haematopoietic stem cells. Nature 2017;545:439–45.

[24] Liang G, Zhou C, Jiang X, Zhang Y, Huang B, Gao S, et al. *De novo* generation of macrophage from placenta-derived hemogenic endothelium. Dev Cell 2021;56:2121–33.e6.

[25] Li Y, Li C, Liu M, Liu S, Liu F, Wang L. The RNA-binding protein CSDE1 promotes hematopoietic stem and progenitor cell generation via translational control of Wnt signaling. Development 2023;150:dev201890.

[26] Lee NYS, Li M, Ang KS, Chen J. Establishing a human bone marrow single cell reference atlas to study ageing and diseases. Front Immunol 2023;14:1127879.

[27] Kasper DM, Hintzen J, Wu Y, Ghersi JJ, Mandl HK, Salinas KE, et al. The N-glycome regulates the endothelial-to-hematopoietic transition. Science 2020;370:1186–91.

[28] Jardine L, Webb S, Goh I, Quiroga Londoño M, Reynolds G, Mather M, et al. Blood and immune development in human fetal bone marrow and Down syndrome. Nature 2021;598:327–31.

[29] Hu C, Zhang N, Hong Y, Tie R, Fan D, Lin A, et al. Single-cell RNA sequencing unveils the hidden powers of zebrafish kidney for generating both hematopoiesis and adaptive antiviral immunity. Elife 2024;13:RP92424.

[30] Han Y, Sun K, Yu S, Qin Y, Zhang Z, Luo J, et al. A Mettl16/m^6^A/mybl2b/Igf2bp1 axis ensures cell cycle progression of embryonic hematopoietic stem and progenitor cells. EMBO J 2024;43:1990–2014.

[31] Han X, Wang R, Zhou Y, Fei L, Sun H, Lai S, et al. Mapping the mouse cell atlas by Microwell-seq. Cell 2018;172:1091–107.e17.

[32] Gao S, Shi Q, Zhang Y, Liang G, Kang Z, Huang B, et al. Identification of HSC/MPP expansion units in fetal liver by single-cell spatiotemporal transcriptomics. Cell Res 2022;32:38–53.

[33] Gao P, Chen C, Howell ED, Li Y, Tober J, Uzun Y, et al. Transcriptional regulatory network controlling the ontogeny of hematopoietic stem cells. Genes Dev 2020;34:950–64.

[34] Fowler JL, Zheng SL, Nguyen A, Chen A, Xiong X, Chai T, et al. Lineage-tracing hematopoietic stem cell origins *in vivo* to efficiently make human HLF+ HOXA+ hematopoietic progenitors from pluripotent stem cells. Dev Cell 2024;59:1110–31.e22.

[35] Ding Y, Wang W, Ma D, Liang G, Kang Z, Xue Y, et al. Smarca5-mediated epigenetic programming facilitates fetal HSPC development in vertebrates. Blood 2021;137:190–202.

[36] Dahlin JS, Hamey FK, Pijuan-Sala B, Shepherd M, Lau WWY, Nestorowa S, et al. A single-cell hematopoietic landscape resolves 8 lineage trajectories and defects in Kit mutant mice. Blood 2018;131:e1–11.

[37] Crosse EI, Gordon-Keylock S, Rybtsov S, Binagui-Casas A, Felchle H, Nnadi NC, et al. Multi-layered spatial transcriptomics identify secretory factors promoting human hematopoietic stem cell development. Cell Stem Cell 2020;27:822–39.e8.

[38] Chen X, Wang P, Qiu H, Zhu Y, Zhang X, Zhang Y, et al. Integrative epigenomic and transcriptomic analysis reveals the requirement of JUNB for hematopoietic fate induction. Nat Commun 2022;13:3131.

[39] Chen C, Yu W, Tober J, Gao P, He B, Lee K, et al. Spatial genome re-organization between fetal and adult hematopoietic stem cells. Cell Rep 2019;29:4200–11.e7.

[40] Calvanese V, Capellera-Garcia S, Ma F, Fares I, Liebscher S, Ng ES, et al. Mapping human haematopoietic stem cells from haemogenic endothelium to birth. Nature 2022;604:534–40.

[41] Bandyopadhyay S, Duffy MP, Ahn KJ, Sussman JH, Pang M, Smith D, et al. Mapping the cellular biogeography of human bone marrow niches using single-cell transcriptomics and proteomic imaging. Cell 2024;187:3120–40.e29.

[42] Baccin C, Al-Sabah J, Velten L, Helbling PM, Grünschläger F, Hernández-Malmierca P, et al. Combined single-cell and spatial transcriptomics reveal the molecular, cellular and spatial bone marrow niche organization. Nat Cell Biol 2020;22:38–48.

[43] The Tabula Muris Consortium. Single-cell transcriptomics of 20 mouse organs creates a Tabula Muris. Nature 2018;562:367–72.

[44] Stuart T, Butler A, Hoffman P, Hafemeister C, Papalexi E, Mauck WM 3rd, et al. Comprehensive integration of single-cell data. Cell 2019;177:1888–902.e21.

[45] Kim D, Paggi JM, Park C, Bennett C, Salzberg SL. Graph-based genome alignment and genotyping with HISAT2 and HISAT-genotype. Nat Biotechnol 2019;37:907–15.

[46] Danecek P, Bonfield JK, Liddle J, Marshall J, Ohan V, Pollard MO, et al. Twelve years of SAMtools and BCFtools. GigaScience 2021;10:giab008.

[47] Anders S, Pyl PT, Huber W. HTSeq — a Python framework to work with high-throughput sequencing data. Bioinformatics 2015;31:166–9.

[48] Granja JM, Corces MR, Pierce SE, Bagdatli ST, Choudhry H, Chang HY, et al. ArchR is a scalable software package for integrative single-cell chromatin accessibility analysis. Nat Genet 2021;53:403–11.

[49] Ramírez F, Dündar F, Diehl S, Grüning BA, Manke T. deepTools: a flexible platform for exploring deep-sequencing data. Nucleic Acids Res 2014;42:W187–91.

[50] Granja JM, Klemm S, McGinnis LM, Kathiria AS, Mezger A, Corces MR, et al. Single-cell multiomic analysis identifies regulatory programs in mixed-phenotype acute leukemia. Nat Biotechnol 2019;37:1458–65.

[51] Haghverdi L, Lun ATL, Morgan MD, Marioni JC. Batch effects in single-cell RNA-sequencing data are corrected by matching mutual nearest neighbors. Nat Biotechnol 2018;36:421–7.

[52] Korsunsky I, Millard N, Fan J, Slowikowski K, Zhang F, Wei K, et al. Fast, sensitive and accurate integration of single-cell data with Harmony. Nat Methods 2019;16:1289–96.

[53] Büttner M, Miao Z, Wolf FA, Teichmann SA, Theis FJ. A test metric for assessing single-cell RNA-seq batch correction. Nat Methods 2019;16:43–9.

[54] Yu G, Wang LG, Han Y, He QY. clusterProfiler: an R package for comparing biological themes among gene clusters. OMICS 2012;16:284–7.

[55] Jin S, Guerrero-Juarez CF, Zhang L, Chang I, Ramos R, Kuan CH, et al. Inference and analysis of cell–cell communication using CellChat. Nat Commun 2021;12:1088.

[56] Tan Y, Cahan P. SingleCellNet: a computational tool to classify single cell RNA-seq data across platforms and across species. Cell Syst 2019;9:207–13.e2.

[57] Zhao S, Zhang A, Zhu H, Wen Z. The ETS transcription factor Spi2 regulates hematopoietic cell development in zebrafish. Development 2022;149:dev200881.

[58] Liu B, Li C, Li Z, Wang D, Ren X, Zhang Z. An entropy-based metric for assessing the purity of single cell populations. Nat Commun 2020;11:3155.

[59] Wu Y, Yang S, Ma J, Chen Z, Song G, Rao D, et al. Spatiotemporal immune landscape of colorectal cancer liver metastasis at single-cell level. Cancer Discov 2022;12:134–53.

[60] Kamimoto K, Stringa B, Hoffmann CM, Jindal K, Solnica-Krezel L, Morris SA. Dissecting cell identity via network inference and *in silico* gene perturbation. Nature 2023;614:742–51.
