# Supplementary material for: HemAtlas: A Multi-omics Hematopoiesis Database
Source: Genomics Proteomics Bioinformatics. 2025 Mar 19;23(2):qzaf026. doi: 10.1093/gpbjnl/qzaf026 (PMC12374576; doi:10.1093/gpbjnl/qzaf026)
Supplement: qzaf026_Supplementary_Data [file qzaf026_supplementary_data.zip › supplementary_material_captions.docx]

## Supplementary material

### File S1 Supplementary method

### Figure S1 The statistics of HemAtlas

**A.** The collected dataset number fraction among different organs/systems in human, mice, zebrafish and HSPCs *in vitro* induction systems. Direct differentiation and direct conversion are two commonly used methods for HSPC *in vitro* induction. Visit <https://ngdc.cncb.ac.cn/hematlas/species/In%20vitro> for more details. PL, placenta. **B.** The statistics of collected dataset number in human, mice, zebrafish and HSPC *in vitro* induction systems. **C.** The dataset number fraction of different sequencing strategies.

### Figure S2 Detailed visualization of selected scRNA-seq and scATAC-seq datasets

**A.** A screenshot showing the detailed description of a selected scRNA-seq dataset (HemSC00000045), in Figure 2C, including the HemAtlas unique ID, a comprehensive description of the omics strategy, the species and organs associated with the dataset, as well as the number of genes and cells included, the reference genome used, and the data standardization method applied. It also provides information on the related publication, including the PMID and NCBI links. Importantly, it details the cell sorting strategy employed for the selected datasets. **B.** Visualization of the zoomed-in webpage for the scRNA-seq dataset (HemSC00000045) shown in Figure 2C. Left: the visualization of selected scRNA-seq data. Cells are colored by their cell-type annotation according to the legend below (red box). The red circle highlights mouse pre-HSCs. pre-HSCs, pre-hematopoietic stem cells. Right: cells are colored by the scaled gene expression of *Myb* according to the legend, with the red circle highlighting the specific expression of *Myb* in mouse pre-HSCs. **C.** Violin plot illustrating the expression levels of *Myb* across various cell types in the selected scRNA-seq dataset. The red box highlights the specific expression of *Myb* in mouse pre-HSCs. **D.** Violin plot showing the TF activity of *Myb* across different cell types in selected scATAC-seq dataset (HemAT00000060). The red box highlights the elevated TF activity of *Myb* in zebrafish HSPCs. VEC, venous endothelial cells; AEC, arterial endothelial cells; HE, hematopoietic endothelial cells.

### Figure S3 Validation of the constructed mouse organ-wide hematopoietic reference

**A.** Visualization of the integration performance of mouse scRNA-seq datasets from different developmental stages using different integration methods. Cells are colored by their original datasets according to the legend. MNN, mutual nearest neighbors. **B.** Box plot displaying the integration local inverse Simpson’s index for each data integration method. Statistical significance between CCA and other integration methods or the no-integration group was assessed using the *t*-test*.* ***, *P* < 0.001. **C.** Expression of *Hlf* (a gene specifically expressed in HSPCs) across different integration results using various strategies in mouse data. Cell colors represent scaled expression levels of *Hlf* according to the legend, with arrows indicating the scatter distribution of *Hlf* expression. **D.** Box plot displaying the rejection rates of the kBET analyses before and after CCA data correction during the construction of mouse cross-stage reference. Statistical significance between the expected and observed rejection rates before and after data correction, as well as between the observed rejection rates before and after correction, was assessed using the *t*-test. ***, *P* < 0.001. kBET, k-nearest neighbor batch effect test.

### Figure S4 Organ-wide hematopoietic references based on scRNA-seq data in zebrafish

**A.** The visualization of zebrafish AGM atlas, which includes 13,987 cells and 13 major cell types. Cells are colored by their cell-type annotation according to the legend. **B.** Dot plot showing the expression of markers genes for cell annotation in zebrafish AGM atlas. The size of the dot corresponds to the percentage of cells expressing the gene. The color represents the average expression level. **C.** The visualization of zebrafish CHT atlas, which includes 27,907 cells and 11 major cell types. Cells are colored by their cell-type annotation according to the legend. **D.** The visualization of zebrafish KM atlas, which includes 21,060 cells and 10 major cell types. Cells are colored by their cell-type annotation according to the legend. **E.** The visualization of zebrafish cross-stage hematopoietic reference, which includes 62,954 cells and 22 major cell types across three developmental stages (AGM, CHT, and KM). Cells are colored by their cell-type annotation according to the legend. **F.** UMAP visualization of the represented zebrafish cell types and scaled expression of the corresponding cell type-specific marker genes in the zebrafish cross-stage hematopoietic reference. Cells are colored by their cell-type annotation and scaled gene expression separately according to the legend. **G.** The visualization of zebrafish cross-stage hematopoietic reference. Cells are colored by the stages to which they belong according to the legend. **H.** The cell number statistics in the zebrafish cross-stage hematopoietic reference across three developmental stages (AGM, CHT, and KM). The color represents corresponding developmental stages according to the legend.

### Figure S5 Organ-wide hematopoietic references based on scRNA-seq data in humans

**A.** The visualization of human AGM atlas, which includes 13,643 cells and 10 major cell types. Cells are colored by their cell-type annotation according to the legend. **B.** The visualization of human FL atlas, which includes 111,907 cells and 17 major cell types. Cells are colored by their cell-type annotation according to the legend. **C.** The visualization of human BM atlas, which includes 117,349 cells and 13 major cell types. Cells are colored by their cell-type annotation according to the legend. **D.** Dot plot showing the expression of markers genes for cell annotation in human BM atlas. The size of the dot corresponds to the percentage of cells expressing the gene. The color represents the average expression level. **E.** The visualization of human cross-stage hematopoietic reference, which includes 242,899 cells and 31 major cell types across three developmental stages (AGM, FL, and BM). Cells are colored by their cell-type annotation according to the legend. **F.** UMAP visualization of the represented human cell types and scaled expression of the corresponding cell type-specific marker genes in the human cross-stage hematopoietic reference. Cells are colored by their cell-type annotation and scaled gene expression separately according to the legend. **G.** The visualization of human cross-stage hematopoietic reference. Cells are colored by the stages to which they belong according to the legend. **H.** The cell number statistics in the human cross-stage hematopoietic reference across three developmental stages (AGM, FL, and BM). The color represents corresponding developmental stages according to the legend.

### Figure S6 The construction of human HSPC classifier using *in vivo* data

**A.** The visualization of HSPCs in the human AGM, FL, and BM stage-specific atlas. **B.** The constructed human HSPC cross-stage atlas as an example. Cells are colored by the corresponding stages and the scaled expression of selected human HSPC marker genes. Mouse and zebrafish HSPC cross-stage cell atlas was constructed with similar methods (File S1). **C.** The precision and recall curve of classification performance showing the HSPC classifier training process in human HSPC cross-stage atlases. **D.** The classification heat map illustrates the classification scores obtained from the trained HSPC classifier in human *in vivo* data. Notably, HSPCs from the same developmental stages exhibit significantly higher classification scores, and the control random group displays elevated scores within its own category. The color represents the classification scores. **E.** The visualization of stage-specific average top pairs genes expression for training data in human *in vivo* data. The color represents the expression of gene pairs. Avg, average. **F.** The attribution plot showing the classification fraction of HSPCs in different categories based on the trained HSPC classifier in humans.

### Figure S7 The HSPC subclusters across stages and species

**A.** The dot plots showing the expression of HSPC marker genes for each species in the constructed cross-stage references in zebrafish (Figure S4E), mice (Figure 3F), and humans (Figure S5E). The size of the dot corresponds to the percentage of cells expressing the gene in each cell type. The color represents the average expression level. Statistical analyses were conducted using a two-sided Wilcoxon rank-sum test to compare the expression levels of HSPC marker genes between HSPC and non-HSPC cell types. ***, *P* < 0.001. **B.** UMAP visualization of mouse HSPCs before and after CCA data integration. Cells are colored by their original datasets/batches according to the legend. **C.** Box plot displaying the rejection rates of the kBET analyses before and after CCA data correction during the construction of mouse HSPC cross-stage atlas. Statistical significance between the expected and observed rejection rates before and after data correction, as well as between the observed rejection rates before and after correction, was assessed using the *t*-test. ***, *P* < 0.001. **D.** Box plot displaying the integration local inverse Simpson’s index for each data integration method. Statistical significance between CCA and other integration method or the no-integration group was assessed using the *t*-test*.* ***, *P* < 0.001. **E.** The numbers of detected homologues genes in all three species based on the homologene R package. See File S1 for the detail of identifying the homologous genes in all three species. **F.** Left: UMAP visualization of zebrafish HSPC subclusters across three stages. Cells are colored by their annotated HSPC subclusters according to the legend. Middle: dot plot showing the expression of markers genes used for annotating zebrafish HSPC subclusters (Figure S7F left). The size of the dot corresponds to the percentage of cells expressing the genes. The color represents the average expression level. Right: the cell number statistics of zebrafish HSPC subclusters across three stages. The color of bar corresponds to the annotated HSPC subclusters (Figure S7F left). **G.** Left: UMAP visualization of mouse HSPC subclusters across stages. Middle: dot plot showing the expression of markers genes used for annotating mouse HSPC subclusters (Figure S7G left). Right: the cell number statistics of mouse HSPC subclusters across three stages. **H.** Left: UMAP visualization of human HSPC subclusters across stages. Middle: dot plot showing the expression of markers genes used for annotating human HSPC subclusters (Figure S7H left). Right: the cell number statistics of human HSPC subclusters across three stages.

### Table S1 Details of collected multi-omics datasets and constructed organ-wide hematopoietic references in HemAtlas

### Table S2 Summary and comparison of HemAtlas with other hematopoietic databases

### Table S3 Differentially expressed genes used for cell annotation in each stage-specific atlas and across-stage reference

### Table S4 The sequences of the qPCR primers used
