# Supplementary figures and images for: HemAtlas: A Multi-omics Hematopoiesis Database
Source: Genomics Proteomics Bioinformatics. 2025 Mar 19;23(2):qzaf026. doi: 10.1093/gpbjnl/qzaf026 (PMC12374576; doi:10.1093/gpbjnl/qzaf026)

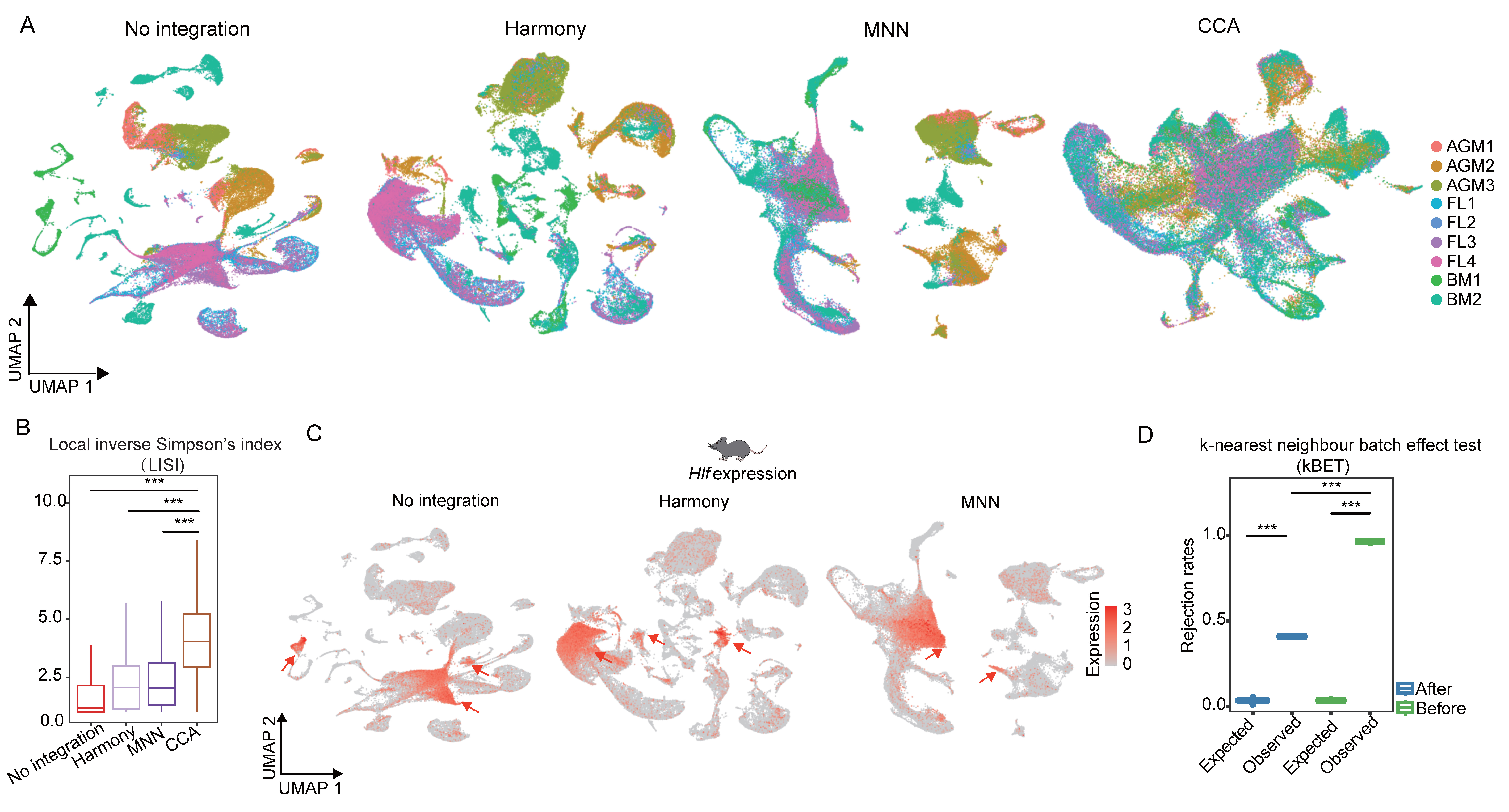

Supplement: qzaf026_Supplementary_Data [file qzaf026_supplementary_data.zip › Figure_S3.jpg]

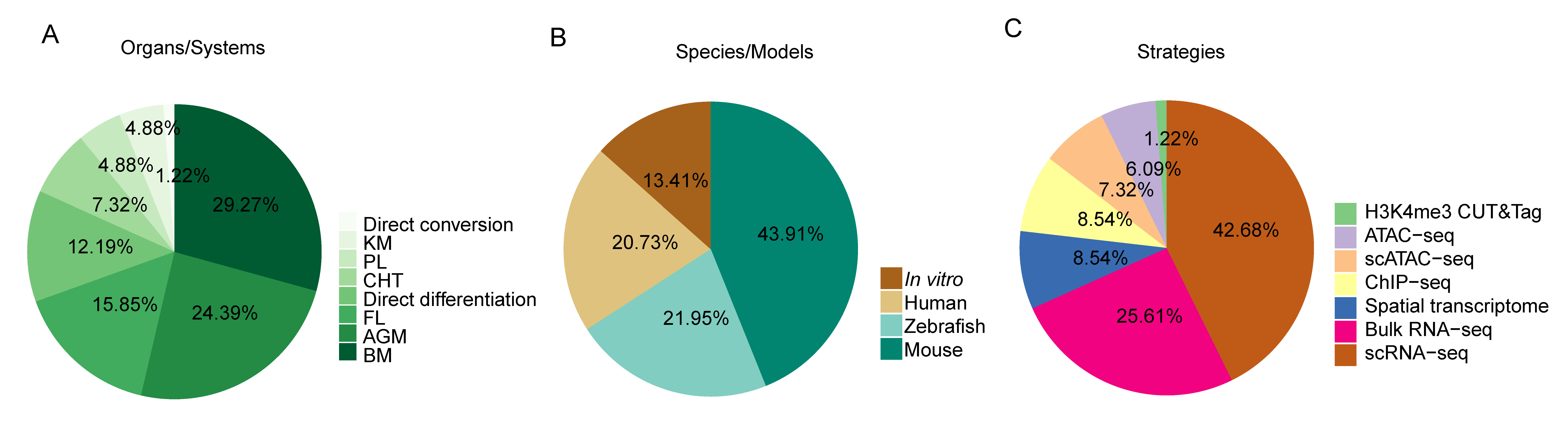

Supplement: qzaf026_Supplementary_Data [file qzaf026_supplementary_data.zip › Figure_S1.jpg]

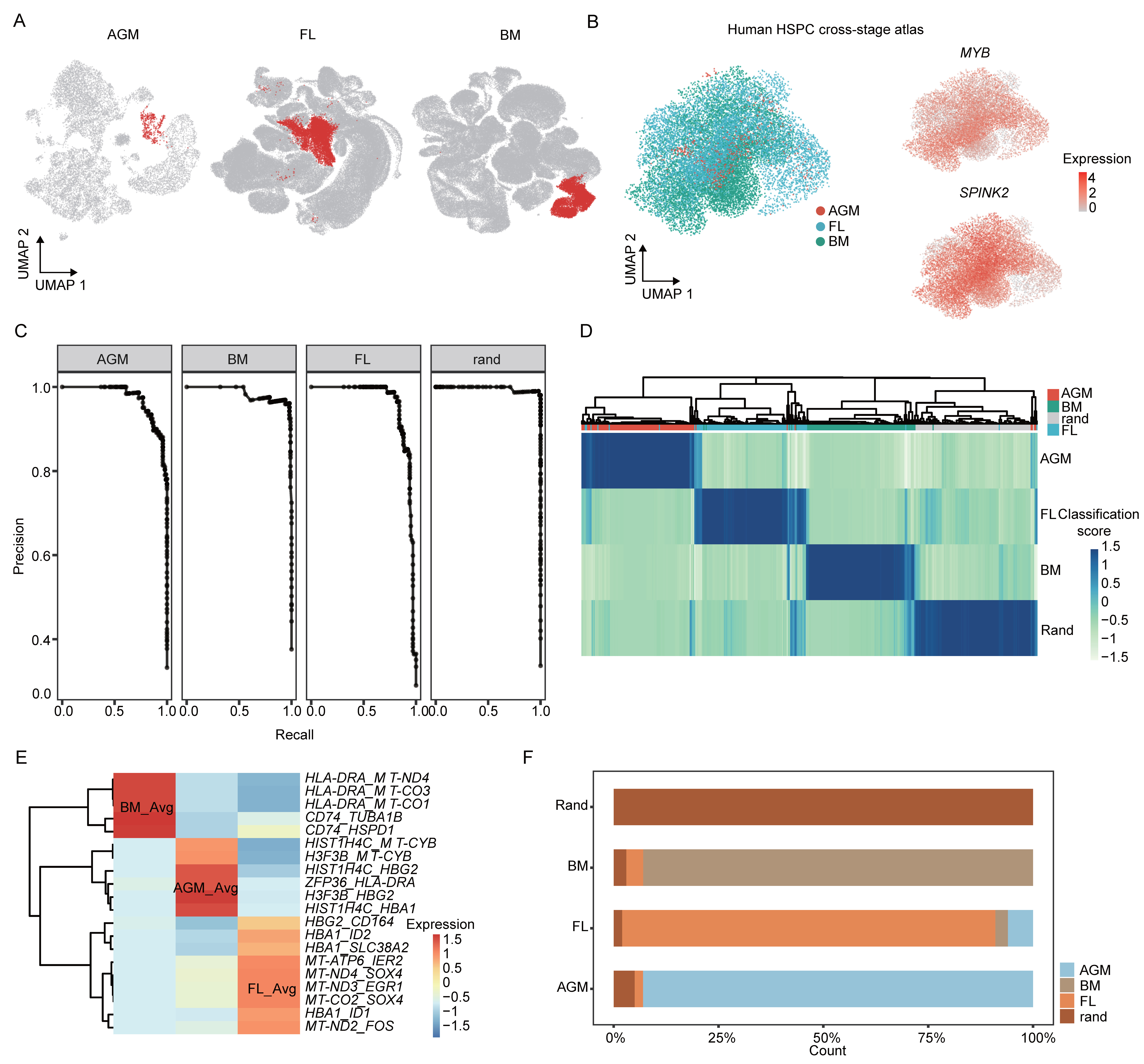

Supplement: qzaf026_Supplementary_Data [file qzaf026_supplementary_data.zip › Figure_S6.jpg]
